# In-vitro characterization of canine multipotent stromal cells isolated from synovium, bone marrow, and adipose tissue: a donor-matched comparative study

**DOI:** 10.1186/s13287-017-0639-6

**Published:** 2017-10-03

**Authors:** Robert N. Bearden, Shannon S. Huggins, Kevin J. Cummings, Roger Smith, Carl A. Gregory, William B. Saunders

**Affiliations:** 1 Department of Small Animal Clinical Sciences, College of Veterinary Medicine and Biomedical Sciences, Texas A&M University, College Station, TX USA; 2 Department of Veterinary Integrative Biosciences, College of Veterinary Medicine and Biomedical Sciences, Texas A&M University, College Station, TX USA; 3 Department of Veterinary Pathobiology, College of Veterinary Medicine and Biomedical Sciences, Texas A&M University, College Station, TX USA; 4 Department of Molecular and Cellular Medicine, Institute for Regenerative Medicine, College of Medicine, Texas A&M University, College Station, TX USA

**Keywords:** Canine, Multipotent stromal cells, Characterization, Differentiation, Immunomodulation, Synovium, Bone marrow, Adipose tissue

## Abstract

**Background:**

The dog represents an excellent large animal model for translational cell-based studies. Importantly, the properties of canine multipotent stromal cells (cMSCs) and the ideal tissue source for specific translational studies have yet to be established. The aim of this study was to characterize cMSCs derived from synovium, bone marrow, and adipose tissue using a donor-matched study design and a comprehensive series of in-vitro characterization, differentiation, and immunomodulation assays.

**Methods:**

Canine MSCs were isolated from five dogs with cranial cruciate ligament rupture. All 15 cMSC preparations were evaluated using colony forming unit (CFU) assays, flow cytometry analysis, RT-PCR for pluripotency-associated genes, proliferation assays, trilineage differentiation assays, and immunomodulation assays. Data were reported as mean ± standard deviation and compared using repeated-measures analysis of variance and Tukey post-hoc test. Significance was established at *p* < 0.05.

**Results:**

All tissue samples produced plastic adherent, spindle-shaped preparations of cMSCs. Cells were negative for CD34, CD45, and STRO-1 and positive for CD9, CD44, and CD90, whereas the degree to which cells were positive for CD105 was variable depending on tissue of origin. Cells were positive for the pluripotency-associated genes *NANOG*, *OCT4*, and *SOX2*. Accounting for donor and tissue sources, there were significant differences in CFU potential, rate of proliferation, trilineage differentiation, and immunomodulatory response. Synovium and marrow cMSCs exhibited superior early osteogenic activity, but when assessing late-stage osteogenesis no significant differences were detected. Interestingly, bone morphogenic protein-2 (BMP-2) supplementation was necessary for early-stage and late-stage osteogenic differentiation, a finding consistent with other canine studies. Additionally, synovium and adipose cMSCs proliferated more rapidly, displayed higher CFU potential, and formed larger aggregates in chondrogenic assays, although proteoglycan and collagen type II staining were subjectively decreased in adipose pellets as compared to synovial and marrow pellets. Lastly, cMSCs derived from all three tissue sources modulated murine macrophage TNF-α and IL-6 levels in a lipopolysaccharide-stimulated coculture assay.

**Conclusions:**

While cMSCs from synovium, marrow, and adipose tissue share a number of similarities, important differences in proliferation and trilineage differentiation exist and should be considered when selecting cMSCs for translational studies. These results and associated methods will prove useful for future translational studies involving the canine model.

**Electronic supplementary material:**

The online version of this article (doi:10.1186/s13287-017-0639-6) contains supplementary material, which is available to authorized users.

## Background

Translation of promising findings from rodent models to humans represents a significant hurdle for cell-based therapies. For this reason, a number of large animal species have been used to bridge the gap from rodents to humans [1–4]. The canine species represents a compelling model for translational studies. When compared to rodents, dogs are large, long-lived, genetically diverse, and share many biochemical and physiological similarities with humans. Canine models have been used successfully to develop adult bone marrow transplantation, gene therapy, and allograft rejection protocols for use in humans [5–7]. Because of their response to learned behaviors such as treadmill exercise, dogs have been used to develop new therapies for cardiovascular and orthopedic diseases [8, 9]. From a biomechanical perspective, the canine skeleton undergoes loading in a manner which approximates that of the human skeleton [10, 11]. For these reasons, canine osteoarthritis, anterior cruciate ligament repair, meniscal injury, and nonunion fracture models are well described [12–19]. Additionally, humans often consider dogs as in-home pets, exposing both to similar environmental stimuli, which helps to eliminate variables between species [20, 21]. For many of these reasons, canine spontaneous diseases have been used to translate novel therapeutics to humans [22, 23]. Benefits of the canine translational model have been described extensively in recent review articles [24, 25].

Multipotent stromal cells (MSCs) are classically isolated from bone marrow and adipose tissues [26–32]; however, recent literature has described the isolation of MSCs from synovium [33], skeletal muscle [34], periosteum [35], and dental pulp [36]. While MSCs isolated from these diverse tissues meet established criteria for MSCs [37], cell proliferation and differentiation vary widely when assessed using established in-vitro assays. These differences may have important implications as investigators consider both the tissue source of MSCs as well as the model species for novel cell-based translational studies.

Although robust literature exists describing synovium, bone marrow, and adipose-derived MSCs in humans, rodents, and other species (a select list of seminal references is provided here) [26–28, 31, 32, 38–50], a more modest number of studies describe the isolation and in-vitro characterization of canine MSCs (cMSCs) from these tissues [29, 30, 34, 51–73]. Unfortunately, drawing comparisons between these studies is difficult due to donor variation, disparate isolation procedures, and the diverse culture and assessment techniques utilized by individual laboratories. Interestingly, results of several studies have demonstrated that cMSCs may respond differently to differentiation protocols established for human MSCs [34, 51, 52, 59, 61, 74–76]. These findings have led some authors to make slight modifications to traditional differentiation protocols in an attempt to improve the consistency of cMSC in-vitro differentiation [30, 34, 52, 53, 75].

In addition to contributing to tissue and organ repair by homing, differentiation, and long-term engraftment, MSCs contribute to tissue repair through production of growth factors and anti-inflammatory cytokines, direct modulation of the immune system, and anti-apoptosis effects [77–84]. In a manner mirroring the human MSC literature, early cMSC characterization studies have typically focused on cell morphology, proliferation, flow cytometry, and trilineage differentiation. Several studies have also reported that canine bone marrow, adipose, and periodontal ligament-derived MSCs are capable of producing growth factors and anti-inflammatory cytokines or directly modulating leukocyte activity [66, 76, 85–89]. However, the immunomodulatory potential of synovium-derived cMSCs has yet to be examined. Moreover, immunomodulatory assays are typically not included in canine donor-matched MSC characterization studies. Thus, a comprehensive report describing the characteristics of donor-matched cMSCs isolated from synovium, bone marrow, and adipose tissues is of the utmost importance to allow investigators interested in the canine model to make informed decisions on cMSC sources for translational studies.

The objective of this study was to comprehensively characterize canine MSCs isolated from synovium, bone marrow, and adipose tissue using a donor-matched study design. Based on work in other species, we hypothesized that canine MSCs isolated from synovium, bone marrow, and adipose tissue would exhibit significant differences in isolation parameters, growth kinetics, colony forming unit (CFU) potential, flow cytometry profiles, trilineage differentiation, and immunomodulatory potential. The results of this study provide insight into important similarities and differences between cMSCs, and will prove useful for investigators considering the canine species for large animal translational studies.

## Methods

For detailed descriptions of all procedures, please refer to Additional file 1.

### Tissue collection and cell isolation

Canine synovium, marrow, and adipose tissue were obtained from four castrated male dogs and one spayed female dog during knee arthroscopy for cranial cruciate ligament rupture (Table 1).

**Table 1:** Signalment, body weight, and body condition score of canine donors

Donor	Age (years)	Sex	Body weight (kg)	BCS (1–9)^a^	Breed
1	2	MC	29.3	4	Catahoula
2	6	MC	71.7	8	Newfoundland
3	3	MC	21.4	4	Mix Breed
4	3	MC	39.4	6	Labrador Retriever
5	5	FS	43.5	6	German Shepherd

Age, sex, body weight (kilograms), body condition score, and breed of the five canine donors enrolled in this study

*MC* male, castrated; *FS* female, spayed; *BCS* body condition score

^a^BCS is a measure of obesity, with 1 representing an extremely thin animal, 4–6 representing an ideal body condition, and 9 representing morbid obesity

Under general anesthesia, marrow aspirates were performed on the proximal humerus. Adipose tissue was obtained from the infrapatellar fat pad prior to arthroscope insertion. Synovium/subsynovial tissues were isolated from the femoropatellar joint during arthroscopy. Sample weights, volumes, and passage 0 (P0) cMSC yields are presented in Table 2.

**Table 2:** Sample yield, colony forming unit potential, and passage 0 yield from five canine donors

Source	Sample weight (g)	Nucleated cell number (×10^3^ cells)/gram of tissue	Colony number/nucleated cells (CFU %)	Mean colony area (mm^2^)	P0 cMSC (×10^6^)/plate
Synovium	0.24 ± 0.01	46.6 ± 62.80	6.48 ± 3.49	0.28 ± 0.03	3.13 ± 3.64
Marrow	4.20 ± 0.80	18.70 ± 28.10	0.01 ± 0.01	0.26 ± 0.13	0.48 ± 0.4
Adipose	0.31 ± 0.10	12.90 ± 12.00	2.63 ± 2.17	0.26 ± 0.13	2.76 ± 5.45

All data reported as mean ± standard deviation

Nucleated cells were isolated using Ficoll™ centrifugation (marrow) or enzymatic digestion (synovium, adipose) and plated at clonal density. Tissue sample weights and the number of nucleated cells recovered from tissue samples adjusted per gram of tissue are presented. After isolation, the colony forming unit (*CFU*) potential of primary cell populations for all 15 donors was performed: 1 × 10^3^ total cells (synovium and adipose) or 4.5 × 10^5^ total cells (marrow) were seeded on 55-cm^2^ plates and incubated for 21 days. Plates were stained with 0.3% crystal violet and the colony number and surface area (mm^2^) were determined for each plate. Using separate MSC isolation plates, the numbers of plastic-adherent canine multipotent stromal cells (cMSCs) recovered at passage 0 (*P0*) after 5–12 days in culture are reported as number of cMSCs/isolation plate

Nucleated cells were isolated from marrow using gradient centrifugation (Ficoll-Paque Plus; GE Health Care Biosciences, Piscataway, NJ, USA) as described previously [90]. Nucleated cells were isolated from adipose and synovial samples using enzymatic digestion [28]. In order to isolate P0 cMSCs, nucleated marrow cells were plated at 3 × 10^4^ cells/cm^2^ in 150-cm^2^ tissue culture dishes in complete culture medium (CCM) containing αMEM, 100 U/ml penicillin, 100 μg/ml streptomycin (Invitrogen), and 10% premium select fetal bovine serum (Atlanta Biological, Inc., Flowery Branch, GA, USA), while nucleated cells from adipose and synovium tissue were plated at 200 cells/cm^2^. Cells were incubated at 37 °C and 5% humidified CO_2_ for 24 hours. For the following 3 days, plates were washed with PBS to remove nonadherent cells followed by media exchange. Culture dishes were subsequently monitored for expansion of the P0 cMSCs with media exchange performed every other day. At 70% confluence (5–12 days), cells were lifted with 0.5% trypsin/EDTA solution (Invitrogen/Thermo Fisher Scientific, Waltham, MA, USA), quantified, and reseeded at 100 cells/cm^2^ for expansion of passage 1 cells. Media were exchanged every other day until cells were 70% confluent. Passage 1 cells were cryopreserved in αMEM with 5% DMSO (Sigma-Aldrich, St. Louis, MO, USA) and 30% FBS in preparation for subsequent experiments. With the exception of the CFU assays in which the initial nucleated cell populations were assayed (specific methods provided below), passage 1 cells were recovered via thawing, plated at 100 cells/cm^2^, and expanded to 70% confluent passage 2 cells for use in experiments.

### CFU assay

The CFU potential of the primary nucleated cell population was determined by plating isolated cells in triplicate on 55-cm^2^ dishes at 4.5 × 10^5^ total cells/dish for marrow tissue and 1 × 10^3^ total cells/dish for synovium and adipose tissue as described previously [48, 91]. At 21 days, plates were stained with 0.3% crystal violet solution (Sigma, St. Louis, MO, USA), washed, photographed, and the colony number determined [92].

### RT-PCR for pluripotency-associated genes

RT-PCR was performed as described previously [30] for canine *GAPDH* [93], *NANOG*, *OCT4*, and *SOX2* [30]. Total RNA was isolated from passage 2 cells and cDNA was synthesized. PCR reactions (20 μl) were performed and products were separated via agarose gel electrophoresis for visualization using Gel Green (Biotium, Hayward, CA, USA).

### Flow cytometry

Passage 2 cMSCs were analyzed with commercially available antibodies, acquired from AbD Serotec (CD9, CD34, CD44, CD45, CD90; Raleigh, NC, USA), Santa Cruz (CD105; Santa Cruz, CA, USA), and R&D Systems (STRO-1; Minneapolis, MN, USA) using a FACSCalibur flow cytometer (BD Biosciences, San Jose, CA, USA), CellQuest acquisition software (BD Biosciences), and FlowJo analysis software (TreeStar Inc., Ashland, OR, USA).

### Proliferation assays

#### Short-term proliferation

To compare the short-term proliferation of synovium, marrow, and adipose cMSCs, cells were plated at 100 cells/cm^2^ in triplicate wells on 12-well tissue culture plates in CCM. Cells were washed with PBS, fixed in 500 μl of DNA quantification buffer at 24-hour intervals for 10 days, and quantified by fluorescence DNA incorporation assay as described previously [94].

#### Long-term proliferation

To compare the proliferation of cMSCs over multiple passages, cells were plated in triplicate at 100 cells/cm^2^ in CCM with media exchange every other day. After 5 days, cells were trypsinized, counted manually, and replated at 100 cells/cm^2^. This process was repeated for a total of five cell passages (25 cumulative days in culture). At each passage, cell yield per plate was determined using a hemocytometer and trypan blue exclusion (*n* = 3 plates/cell preparation) and data were reported as the number of population doublings per passage as described previously [95].

#### Adipogenesis

Canine MSCs were plated at 2 × 10^4^ cells/cm^2^ in 12-well plates (*n* = 4 wells/condition) and were treated with control medium (CCM) or modified adipogenic medium (αMEM containing 1 nM dexamethasone (Sigma), 5 mM rosiglitazone (Sigma), 50 mM pantothenate (Enzo Life Sciences, Farmingdale, NY, USA), 10 mM insulin (Sigma), 30 mM biotin (Enzo), 50 mM isobutylmethylxanthine (Sigma), and 10% serum (5% FBS, 5% rabbit serum; Atlanta Biological)) [30, 34, 53, 74, 96–98]. After 21 days, cells were washed, fixed in 10% neutral buffered formalin, and stained with 0.5% Oil Red O (Sigma). Cells were photographed prior to extraction of Oil Red O for quantification as described previously [96].

#### Early assay of osteogenesis—alkaline phosphatase activity

Canine MSCs were plated at 5 × 10^3^ cells/cm^2^ in 12-well plates (*n* = 3 wells/condition) and were treated with control medium (CCM) or osteogenic basal medium (OBM) (αMEM containing 5% FBS, 10 μg/ml beta-glycerophosphate (Sigma), 50 mg/ml ascorbate-2-phosphate (Sigma)) optimized for cMSCs [94]. In addition, cells were treated with OBM supplemented with 50 or 100 ng/ml of recombinant human bone morphogenic protein-2 (rhBMP-2; R&D Systems) [52, 94]. After 7 days, alkaline phosphatase (ALP) activity was determined as described previously [94]. ALP activity of each well was normalized to the number of cells per well using DNA quantification.

#### Late-stage assay of osteogenesis—Alizarin Red stain mineralization

Detachment of high-density monolayers from polystyrene tissue culture plastic in late-stage mineralization assays is a phenomenon that is not uncommon in cMSC mineralization assays. We identified this phenomenon in our early workings with cMSCs (unpublished observations) and this problem has been reported in prior cMSC literature [30, 34, 51, 52, 61, 74, 75]. In order to prevent monolayer detachment, cMSCs were plated in CCM at 2 × 10^4^ cells/cm^2^ in 12-well plates (*n* = 4 wells/condition). Prior to plating, the periphery of each well was scored mechanically using a sterile stone dremel bit and associated hand chuck in order to create a circular etching around the well margin. Wells were coated with human fibroblast-derived fibronectin (Sigma) at 5 mg/ml in PBS for 30 minutes at 37 °C. Excess fibronectin was removed and cells were seeded in each well. The following day, cells were treated with CCM, OBM, or OBM supplemented with 200 ng/ml rhBMP-2. Media were exchanged twice weekly. After 7 days, 1 nM dexamethasone was added to OBM and OBM + BMP-2 wells, creating osteogenic differentiation media (ODM) to induce mineralization [94, 97]. At 21 days, cells were washed with PBS and fixed in 500 μl of 10% neutral buffered formalin prior to staining in 40 nM Alizarin Red stain (ARS; Sigma) to visualize calcium deposition within osteogenic monolayers. Wells were photographed prior to extraction of ARS for semi-quantification via spectrophotometry using an acetic acid extraction technique as described previously [94].

#### Chondrogenesis

Micromass cultures of cMSCs were generated from 5 × 10^5^ cells using techniques described previously [96, 97, 99]. Chondrogenesis was assessed using digital morphometry to quantify the pellet size, toluidine blue histology, and collagen type II immunohistochemistry. For histologic studies, formalin-fixed pellets were sectioned in paraffin, processed by standard methods, and stained with 1% toluidine blue/sodium borate. Visualization of collagen type II expression was achieved by immunohistochemistry using the Vectastain ABC Kit (Vector Laboratories Inc., Burlingame, CA, USA) and a commercially available rabbit anti-collagen type II antibody (Abcam, Cambridge, MA, USA) as described previously [100, 101].

### Immunomodulation

To assess macrophage-mediated immunomodulation, mouse macrophage cells (RAW 264.7 cell line, American Type Culture Collection TIB-71) were seeded at 1 × 10^4^ cells/cm^2^ in 12-well plates in CCM. After 24 hours, cMSCs were titrated (1 × 10^3^, 1 × 10^4^, 2.5 × 10^4^, and 5 × 10^4^ cells/well) to initiate a 24 hour coculture (*n* = 3 replicates/cMSC dosage). Lipopolysaccharide (LPS) (*Escherichia coli* 055:B5 strain; Sigma) was introduced to each well at 0.5 μg/ml to induce macrophage activation. Cocultures were allowed to respond for 18 hours and conditioned media were collected and stored at –20 °C. Media were thawed on ice and analyzed for murine TNF-α (DY410-05) and IL-6 (DY406-05) protein concentrations via enzyme-linked immunosorbent assay (ELISA) according to the manufacturer’s protocol (R&D Systems).

### Statistical analysis

Descriptive statistics were generated using GraphPad Prism 6.0 (GraphPad Software, La Jolla, CA, USA) and reported as mean ± standard deviation (SD). Data were imported into a commercial statistical software program (SAS version 9.4; SAS Institute Inc., Cary, NC, USA) for inferential statistics. Repeated-measures ANOVA was used to determine whether each parameter differed significantly by tissue type and treatment group, as appropriate, with donor dog regarded as a random effect. The Tukey method was used to adjust for multiple pairwise comparisons. For all analyses, *p* < 0.05 was considered significant.

## Results

### Cell isolation and CFU potential

**Fig. 1:**
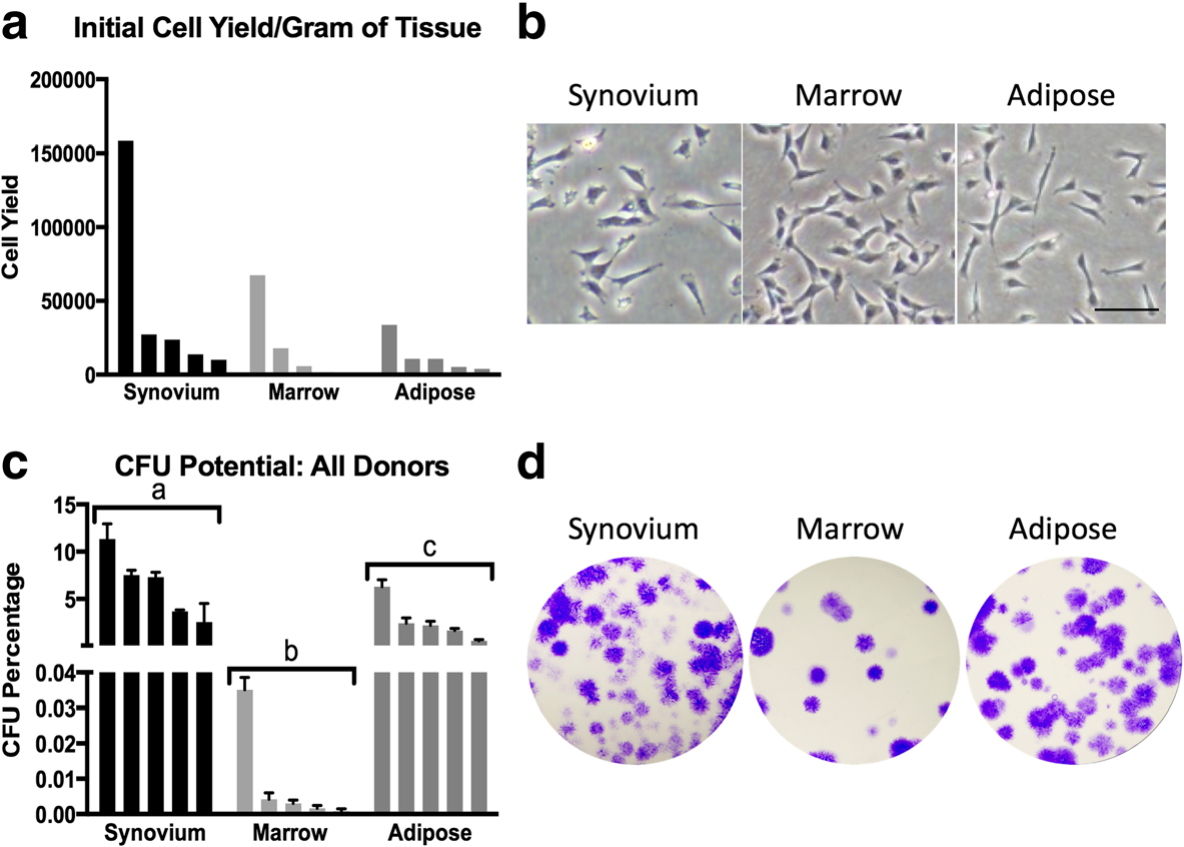
Initial cell isolation and colony forming unit (*CFU*) potential. Synovium, marrow, and adipose tissues were obtained from five canine donors presenting for rupture of the cranial cruciate ligament. Cells were isolated using Ficoll™ centrifugation (marrow) or enzymatic digestion (synovium, adipose) and plated at clonal density. **a** Initial nucleated cell yield for all 15 donors, normalized to tissue weight. **b** Representative 10× objective phase-contrast microscopy images 7 days post isolation (*bar* = 100 μm). **c** CFU potential of primary cell populations for all 15 donors: 1 × 10^3^ total cells (synovium and adipose) or 4.5 × 10^5^ total cells (marrow) were seeded on 55-cm^2^ plates and incubated for 21 days. Plates were stained with 0.3% crystal violet and colony counts were performed on each plate. CFU potential is defined as the number of colonies present divided by total number of seeded cells, expressed as a percentage of the total seeded cells. Data reported as mean ± SD (*n* = 3 plates/tissue). *a*, *b*, *c* denote significant differences between tissue sources of cMSCs (*p* < 0.0001). **d** Photographs of CFU plates from a single representative donor. For **a** and **c**, data are reported in descending order for each tissue type

Mononuclear cells were successfully isolated from each donor and tissue sample (Table 2). Synovium (46.6 × 10^3^ ± 62.8 × 10^3^ cells/g of tissue) provided greater numbers of nucleated cells when compared to the marrow (18.7 × 10^3^ ± 28.1 × 10^3^ cells/g of tissue) and adipose tissue (12.9 × 10^3^ ± 12.0 × 10^3^ cells/g of tissue), although these differences were not significant (*p* = 0.2). In addition, we were unable to detect a difference in the number of total nucleated cells isolated from marrow or adipose tissue (*p* = 0.8) (Fig. 1a, Table 2). After 5–14 days of culture, cMSCs were identified in primary expansion plates as plastic-adherent, spindle-shaped cells (Fig. 1b). All primary nucleated cell populations obtained from the 15 tissue samples exhibited some degree of colony forming unit (CFU) potential. Using repeated-measures ANOVA, significant differences were observed in the CFU potential between the three tissues (*p* < 0.0001), with synovium (6.48 ± 3.49%) and adipose (2.63 ± 2.17%) tissue exhibiting markedly higher CFU potential when compared to marrow tissue (0.009 ± 0.01%). Specifically, the CFU potential of synovium cMSCs was significantly greater than that of both adipose-derived (*p* < 0.001) and marrow-derived (*p* < 0.0001) cells, whereas the CFU potential of adipose cMSCs was significantly greater than that of marrow-derived cells (*p* < 0.01) (Fig. 1c, d, Table 2). These CFU values are consistent with studies reporting CFU potential of human MSCs derived from synovium, marrow, and adipose tissue.

### Cell surface marker expression

**Fig. 2:**
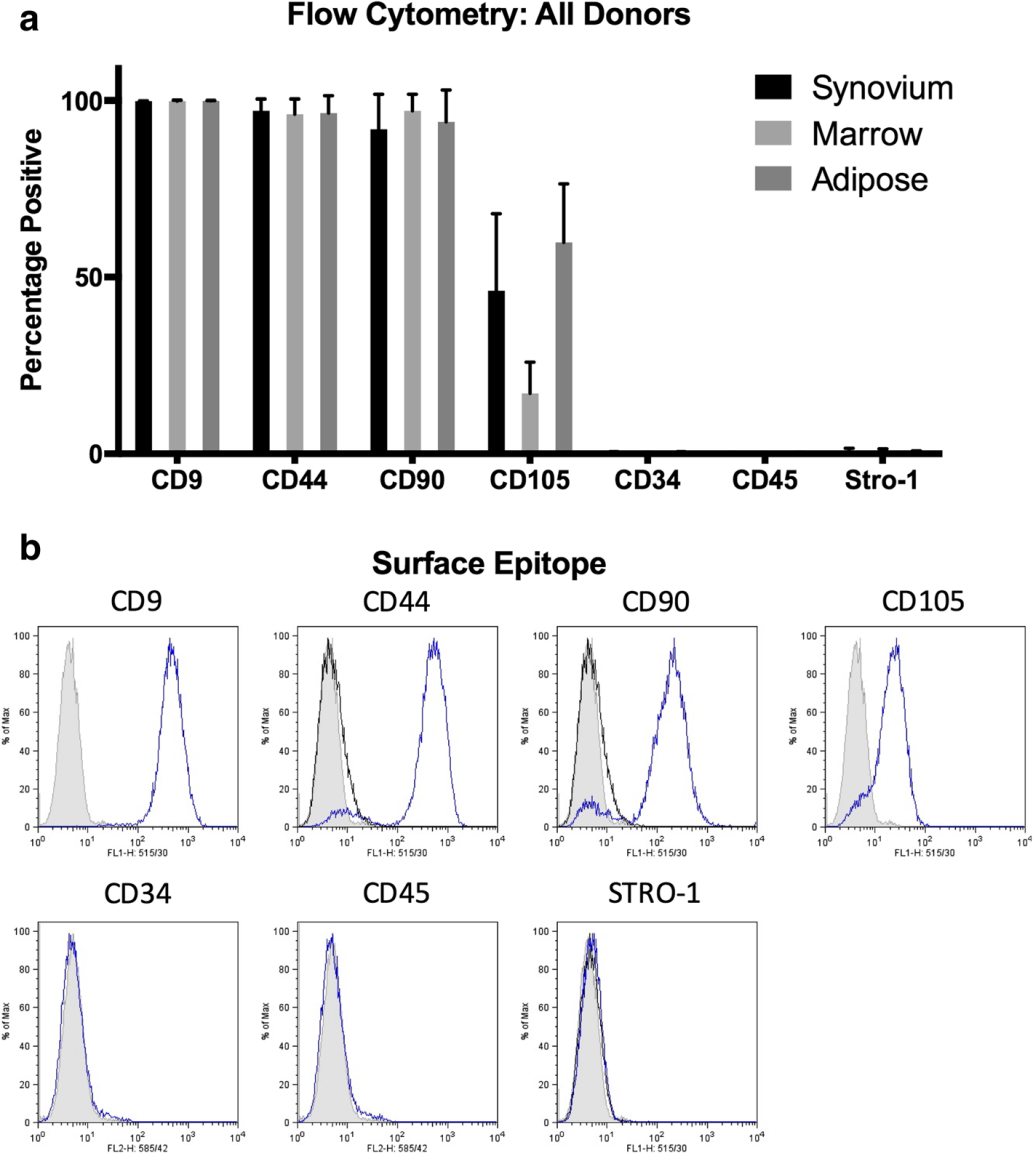
Flow cytometry for synovium, marrow, and adipose cMSCs. **a** Percentage of positive cells reported as mean ± SD for synovium, marrow, and adipose cMSCs isolated from five canine donors. **b** Representative histograms demonstrating positive and negative staining of marrow cMSCs from a single donor

A flow cytometry panel capable of cross-reacting with canine isoforms of cell surface markers was used to characterize each preparation of cMSCs. Representative flow cytometry results for a preparation of MSCs derived from canine bone marrow are shown in Fig. 2. The mean ± SD antibody labeling results for all 15 preparations of cMSCs is provided in Additional file 2. All cMSCs were negative for the leukocyte markers CD34 and CD45. Cells were consistently positive for CD9, CD44, and CD90. Interestingly, there was variable staining for CD105 (Endoglin), with synovium (46.16 ± 21.78%) and adipose (59.84 ± 15.57%) cMSCs exhibiting higher percentage positive staining cells when compared to marrow cMSCs (17.12 ± 8.85%). Additionally, cMSCs were negative for STRO-1, despite confirming cross-reactivity of the commercially available STRO-1 antibody on canine peripheral blood (data not shown).

### Pluripotency-associated gene expression

**Fig. 3:**
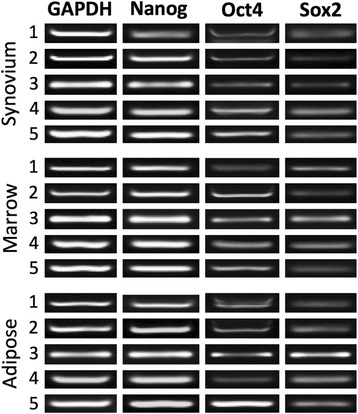
Expression of pluripotency-associated genes in synovium, marrow, and adipose cMSCs. Passage 2 cMSCs were qualitatively evaluated for the pluripotency-associated genes *NANOG*, *OCT4*, and *SOX2* using RT-PCR. All 15 cMSC cell preparations were positive when assessed using RT-PCR. Representative images of *NANOG* (274 bp), *OCT4* (141 bp), and *SOX2* (142 bp) gene expression from synovium, marrow, and adipose-derived cMSCs of a single donor are shown. Canine *GAPDH* was used as a housekeeping gene. *bp* base pairs

Reverse-transcription PCR was used to evaluate all cell preparations for the canine isoforms of the pluripotency associated transcription factors *NANOG*, *OCT4*, and *SOX2* [30]. All cMSC preparations were positive for each gene (Fig. 3).

### Proliferation assays

**Fig. 4:**
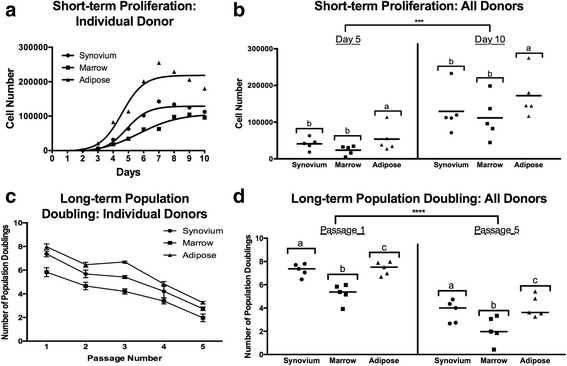
Short-term and long-term proliferation of cMSCs. Passage 2 cMSCs were seeded at 100 cells/cm^2^ in CCM on 12-well plates (*n* = 3 wells/cell preparation) with media exchange every other day. Mean cell number determined daily for 10 consecutive days using DNA quantification. **a** Mean short-term proliferation of a representative donor, demonstrating higher proliferation rates of adipose and synovium cMSCs. **b** Scatter plot demonstrating the number of cMSCs for all 15 cell preparations at days 5 and 10. Each data point represents the cell number for an individual cMSC preparation (*bar* = mean cell yield across the five donors). Long-term proliferation was determined over a five-passage, 25-day time course ﻿﻿as described in the Methods section﻿﻿﻿. **c** Population doubling (mean ± SD) of a representative donor, demonstrating increased doubling rate of adipose and synovium cMSCs, particularly at passages 1–3. **d** Population-doubling scatter plot for all 15 cMSC preparations at passages 1 and 5. Each data point represents the population-doubling rate for an individual cMSC preparation (*bar* = mean population doubling rate across the five donors). Note: **b, d** significant differences in cell number: ***p < 0.001, ****p < 0.0001. *a, b, c* denote significant differences between tissue sources (*p* < 0.05)

Both short-term and long-term proliferation assays were used to assess the proliferation of donor-matched cMSCs derived from synovium, marrow, and adipose tissues. In short-term assays, there were significant differences in proliferation between synovium, marrow, and adipose cMSCs (*p* < 0.01). Consistent with a previous study evaluating human MSCs [28], adipose and synovium cMSCs proliferated more rapidly than marrow cMSCs. A proliferation curve from a representative donor is shown (Fig. 4a). Scatter plots reporting the number of recovered cells at day 5 and day 10 for all 15 cMSC isolates are also provided (Fig. 4b). Using repeated-measures ANOVA, adipose cMSC proliferation was significantly greater than that of marrow (*p* < 0.01) and synovium (*p* < 0.05). While synovium cMSCs tended to proliferate more rapidly than marrow cMSCs, differences were not significant in the short-term assay (*p* = 0.2). These results indicate that when using a donor-matched study design, the tissue source of cMSCs affects short-term proliferation of these cells.

In long-term assays, the tissue source of cMSCs had a significant effect on the number of recovered cells over the five-passage, 25-day time course (*p* < 0.0001) (Fig. 4c, d). As reported previously in other species, population doubling decreased significantly with sequential passaging (*p* < 0.0001). Results for a representative donor are shown in Fig. 4c. The mean population doubling at passages 1 and 5 for all cMSC preparations is also provided (Fig. 4d). Using repeated-measures ANOVA, population doubling of both adipose (*p* < 0.0001) and synovium (*p* < 0.0001) cMSCs was significantly greater than that of marrow cMSCs across all passages. Additionally, the population doubling of adipose and synovium cMSCs was significantly different (*p* = 0.02). These results demonstrate the greater proliferation abilities of adipose and synovium cMSCs as well as the finite proliferation of cMSCs derived from all three tissues.

### Trilineage differentiation

#### Adipogenesis

**Fig. 5:**
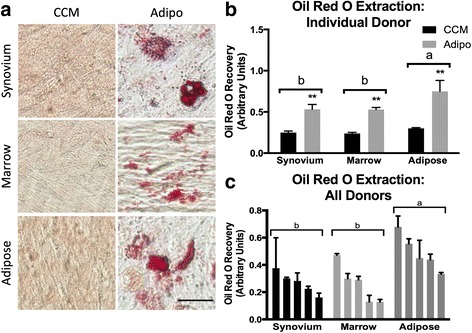
Adipogenesis of synovium, marrow, and adipose of cMSCs. **a** Passage 2 cMSCs were cultured in quadruplicate wells in CCM or modified adipogenic media with media exchange twice weekly. At 21 days, cells were formalin fixed and evaluated for lipid accumulation with Oil Red O (*bar* = 25 μm). **b** Oil *Red* O quantification (mean ± SD) for a representative donor. **Significantly different Oil *Red* O quantification between treatment conditions (*p* < 0.01). **c** Mean ± SD Oil *Red* O quantification for all 15 cMSC isolates. CCM values have been subtracted from adipogenic values to facilitate data presentation. Data are reported in descending order for each tissue. For **b** and **c**, *a*, *b* denote significant differences between tissue sources of cMSCs (*p* < 0.001). *CCM* complete culture medium

Adipogenesis was evaluated at 21 days by both visual assessment of lipid vacuole accumulation and quantification of Oil Red O staining. All cMSCs underwent varying degrees of adipogenesis, with increased vacuole formation and Oil Red O staining when compared to control (CCM). Oil Red O accumulation for a representative donor is shown in Fig. 5a. Morphologically, synovium and adipose cMSCs produced medium to large, grape-like vacuoles, whereas marrow cMSCs produced small, diffuse vacuoles. When evaluating Oil Red O extraction for Fig. 5a, significant differences were detected (Fig. 5b). Oil Red O extraction values for all 15 cMSC preparations are provided (Fig. 5c). Using repeated-measures ANOVA, there was a significant increase in Oil Red O extraction for cMSCs treated with adipogenic media as compared to CCM control wells (*p* < 0.0001, results not shown). In addition, there were significant differences in Oil Red O extraction based on tissue source of the cMSCs (*p* < 0.001). Adipose cMSCs had significantly greater Oil Red O extraction values across all donors when compared to marrow (*p* < 0.01) and synovium (*p* < 0.001) cMSCs; however, we were unable to detect a difference in Oil Red O extraction between synovium and marrow cMSCs (*p* = 0.4). These results indicate that while synovium, marrow, and adipose cMSCs are capable of undergoing adipogenesis, adipose cMSCs are superior in their adipogenic ability.

### Early osteogenesis—ALP activity

**Fig. 6:**
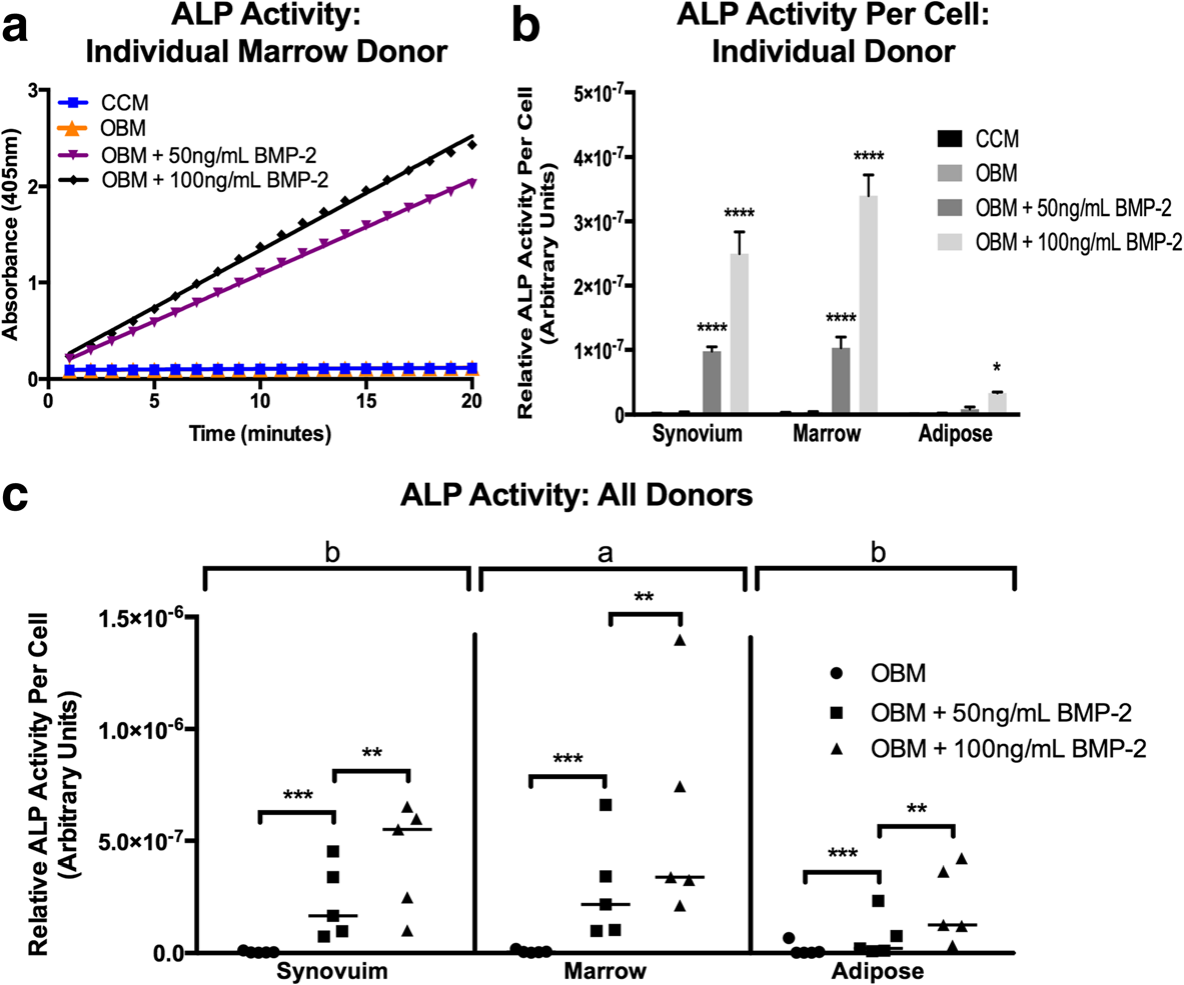
Early osteogenesis of synovium, marrow, and adipose cMSCs. Early osteogenesis was determined using the alkaline phosphatase (*ALP*) activity assay. Passage 2 cMSCs were cultured in CCM, OBM, or OBM + rhBMP-2 for 7 days and evaluated for the ability to convert the colorless substrate PNPP to colorimetric PNP over time. **a** Kinetic ALP activity results for a single cMSC preparation from a representative donor. ALP activity was determined by spectrophotometer (absorbance 405 nM) over a 20-minute time course. **b** ALP activity normalized to cell number by DNA quantification for synovium, marrow, and adipose cMSCs from a representative donor, demonstrating the minimal response of adipose cMSCs to OBM or OBM containing rhBMP-2. **c** Scatter plots demonstrating ALP activity for all 15 cMSC preparations organized by tissue and media condition. Each data point represents the ALP activity per cell for an individual cMSC preparation and a given media condition (*bar* = mean across the five donors). For **b** and **c**, significant differences between treatment groups: *p < 0.05, **p < 0.01, ***p < 0.001, ****p < 0.0001. *a*, *b* denote significant differences between tissue sources of cMSCs (*p* < 0.01). *BMP-2* bone morphogenic protein-2, *CCM* complete culture medium, *OBM* osteogenic basal medium

Early osteogenesis was evaluated at 7 days using the ALP activity assay. In contrast to MSCs from other species, it has been reported previously that cMSCs require osteogenic medium supplemented with BMP-2 in order to exhibit robust ALP activity [52, 75]. In order to confirm this unique property of cMSCs isolated from synovium, marrow, and adipose tissue, ALP activity assays were performed on all 15 cMSC isolates after initiation of osteogenesis with OBM or OBM containing 50 or 100 ng/ml rhBMP-2. Over the 20-minute kinetic assay, there was no detectable ALP activity in cMSCs treated with CCM or OBM (a medium known to induce robust ALP activity in human MSCs); however, OBM containing rhBMP-2 induced a dose-dependent increase in ALP activity. Results from a representative donor (marrow cMSCs) are shown in Fig. 6a. ALP activity, normalized to a per-cell basis using DNA quantification, for synovium, marrow, and adipose cMSCs from the donor presented in Fig. 6a is also provided (Fig. 6b).

ALP activity values for all 15 cMSCs are shown in Fig. 6c. Using repeated-measures ANOVA, there were significant differences in ALP activity based on media condition (*p* < 0.0001) as well as the cMSC tissue source (*p* < 0.01). There was no detectable difference in ALP activity between cMSCs cultured in CCM or OBM (*p* = 0.999). ALP activity was significantly increased for cMSCs treated with OBM + 50 ng/ml rhBMP-2 (*p* < 0.001) and OBM + 100 ng/ml rhBMP-2 (*p* < 0.0001) when compared to CCM or OBM. Additionally, cMSCs treated with OBM + 100 ng/ml rhBMP-2 exhibited significantly higher ALP activity as compared to cMSCs treated with OBM + 50 ng/ml rhBMP-2 (*p* < 0.001). When considering the cMSC tissue source, marrow-derived cMSCs exhibited significantly greater ALP activity when compared to adipose cMSCs (*p* < 0.001) and synovium cMSCs (*p* < 0.05), while no significant difference in ALP activity was observed between synovium and adipose cMSCs (*p* = 0.1). Collectively, these data demonstrate that cMSCs require exogenous BMP-2 to exhibit detectable ALP activity, and that rhBMP-2 supplementation drives ALP activity in a dose-dependent manner. Moreover, while synovium and marrow cMSCs from individual donors respond robustly to OBM + rhBMP-2, donor-matched adipose-derived cMSCs exhibit markedly reduced early osteogenic differentiation when assessed by the ALP activity assay.

#### Late-stage osteogenesis—ARS mineralization assay

**Fig. 7:**
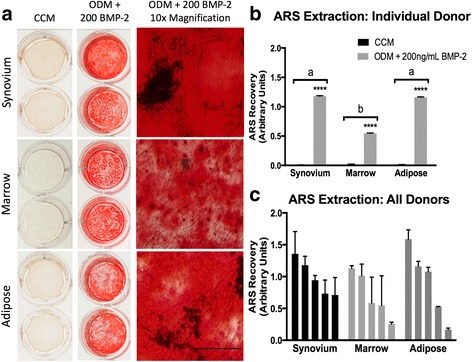
Late-stage osteogenesis of synovium, marrow, and adipose cMSCs. Passage 2 cMSCs were cultured in triplicate wells in CCM or ODM with media exchange twice weekly. **a** After 21 days of culture in CCM (*left column*) or ODM + 200 ng/ml of rhBMP-2 (*middle and right columns*) monolayers were fixed in 10% formalin and stained with ARS. Plates were photographed (*left and middle columns*) and imaged with 10× objective light microscopy (*right column*) to document ARS accumulation (*bar* = 125 μm). **b** ARS extraction (mean ± SD) for a representative donor. ****Significant differences between treatment groups (*p* < 0.0001). **c** Mean ± SD ARS extraction values for all 15 cMSC preparations. CCM values were subtracted from osteogenic values to facilitate presentation of results. Data are reported in descending order for each tissue. For **b** and **c**, *a*, *b* denote significant differences between tissue sources of cMSCs, if present (*p* < 0.0001). *ARS* Alizarin Red stain, *BMP-2* bone morphogenic protein-2, *CCM* complete culture medium, *ODM* osteogenic differentiation medium

Late-stage, biomineralizing osteogenesis was evaluated at 21 days with visual staining of calcium deposits within monolayers and semi-quantification of extracted ARS. All cMSCs underwent varying degrees of osteogenesis as assessed by ARS binding (Fig. 7). ARS results for a representative donor are shown in Fig. 7a. While ARS did not accumulate in control wells (CCM) or in wells treated with ODM lacking BMP-2 (results not shown), ARS accumulation was robust in synovium, marrow, and adipose cMSCs treated with ODM + 200 ng/ml rhBMP-2. Semi-quantification of ARS extraction for this donor is shown in Fig. 7b. There was a significant increase in ARS extraction for all three tissues when compared to control (CCM), with synovium and adipose cMSCs exhibiting significantly greater ARS extraction when compared to marrow cMSCs for this individual donor.

ARS extraction values for all 15 cMSC cell lines are provided (Fig. 7c). Using repeated-measures ANOVA, there was a significant increase in ARS extraction in cMSCs treated with ODM + 200 ng/ml rhBMP-2 as compared to control (*p* < 0.0001). However, when accounting for variation across donors, there was no significant difference in ARS extraction based on the tissue source of the cMSCs (*p* = 0.5).

### Chondrogenesis

**Fig. 8:**
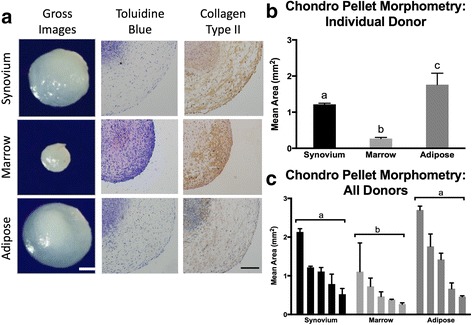
Chondrogenesis of synovium, marrow, and adipose cMSCs. Passage 2 cMSCs were evaluated for chondrogenesis using the micromass pellet technique: 5 × 10^7^ cells from each cMSC preparation were pelleted in triplicate and incubated for 21 days in chondrogenic medium with media exchange twice weekly. **a** Pellets were photographed (gross images, *bar* = 300 μm), formalin fixed, and sectioned for histology. Pellets were positive for proteoglycan (toluidine blue) and collagen type II (10× objective, *bar* = 150 μm), although the intensity of staining varied across donor and tissue source of cMSC. **b** Pellet morphometry for a representative donor (mean ± SD). **c** Mean ± SD pellet area (mm^2^) of chondrogenic pellets for all 15 cMSC preparations. Data are reported in descending order for each tissue. For **b** and **c**, *a*, *b*, *c* denote significant differences between tissue source of cMSCs (*p* < 0.01)

Each of the 15 preparations of cMSCs underwent condensation and adopted a spherical translucent appearance in response to chondrogenic differentiation medium. Furthermore, cell pellets increased in size over 21 days. Results from a representative donor are shown in Fig. 8a,b. Synovium and adipose cMSCs produced larger pellets as compared to marrow cMSCs. Marrow cMSCs were smaller in size and consistently displayed deep staining for toluidine blue and collagen type II (Fig. 8a). Although adipose cMSCs produced the largest pellets for this donor, toluidine blue and collagen type II immunohistochemistry staining was apparent within the center of the pellet, whereas much of the periphery of the pellet exhibited limited toluidine blue or collagen type II staining. The synovial pellet for this same donor demonstrated more uniform collagen type II staining throughout the pellet, while toluidine blue staining was limited to the center of the structure. These subjective appearances were consistent across the 15 preparations of cMSCs.

Digital morphometric results for all 15 cMSC isolates treated with chondrogenic differentiation medium for 21 days are shown in Fig. 8c. Using repeated-measures ANOVA, there was a significant difference in pellet size based on the cMSC tissue of origin (*p* < 0.01). Both adipose (*p* < 0.001) and synovium (*p* < 0.01) cMSC pellets were significantly larger than marrow cMSC pellets. We were unable to detect a significant difference in pellet size when comparing synovium or adipose cMSCs (*p* = 0.2). As already described, when subjectively assessing toluidine blue and collagen type II stain accumulation, marrow cMSC pellets exhibited deeper and more uniformly consistent staining compared to synovium and adipose-derived cMSC pellets. Synovium cMSCs exhibited increased collagen type II staining throughout the sections, whereas adipose-derived cMSC collagen type II staining was markedly reduced in the periphery of each section.

### Immunomodulation

**Fig. 9:**
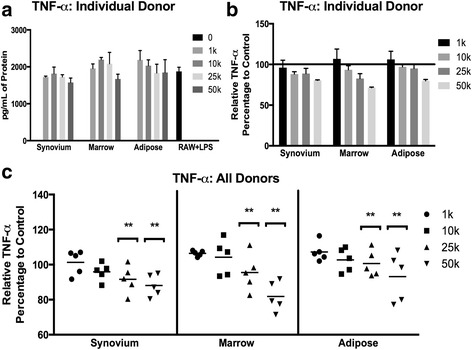
Immunomodulation of murine TNF-α by synovium, marrow, and adipose cMSCs. Passage 2 cMSCs (1 × 10^3^–50 × 10^3^) were cocultured with 1 × 10^4^ murine macrophage cells in 12-well plates in CCM (*n* = 3 wells/condition). After 24 hours, LPS (0.5 μg/ml) was added to cocultures to activate macrophages. After 18 hours, media were collected and ELISA performed to determine the concentration of secreted murine TNF-α. **a** Representative murine TNF-α concentrations (mean ± SD) for an individual donor. *RAW + LPS* denotes TNF-α concentration from murine macrophages (RAW cells) in the absence of cMSCs (positive control). **b** Data from **a** were transformed to reflect the percentage change in TNF-α relative to the RAW + LPS positive control in preparation for comparative analysis across all 15 cMSC preparations, reported as mean ± SD. **c** Scatter plots demonstrating the percentage change of TNF-α concentration relative to positive control for all 15 cMSC preparations, organized by tissue and number of cMSCs present within cocultures. Each data point represents the relative murine TNF-α for an individual cell preparation and “dose” of cMSC (*bar* = mean across the five donors). For all three tissues, TNF-α concentrations decreased in response to increasing numbers of cocultured cMSCs. Significant differences between numbers of cocultured cMSCs: **p < 0.01. *LPS* lipopolysaccharide, *TNF-α* tumor necrosis factor alpha

To assess the immunomodulatory potential of cMSCs, we established macrophage and cMSC coculture experiments in which a murine macrophage cell line (RAW 264.7 cell line) was cultured alone or in combination with increasing numbers of cMSCs. Cultures were challenged with LPS and assessed for secreted murine TNF-α and IL-6 concentrations were determined in conditioned media to measure inflammatory responses by the RAW cells and also to assess whether cMSCs could affect the production of these two cytokines. The concentration of TNF-α detected in a representative coculture experiment is reported (Fig. 9a). As has been described in other species [102, 103], cMSC coculture resulted in a dose-dependent decrease in the concentration of murine TNF-α. In order to make direct comparisons across all 15 cMSC preparations, the measured concentrations of TNF-α were normalized to the positive control (murine cells alone stimulated with LPS) for each assay and reported as the percentage TNF-α relative to control (Fig. 9b).

The relative concentrations of TNF-α for all 15 cMSC coculture experiments are shown in Fig. 9c. Using repeated-measures ANOVA, there were significant differences in TNF-α based on the number of cMSCs present within the cocultures (*p* < 0.0001), whereas the tissue source of cMSCs had no effect on TNF-α (*p* = 0.5). TNF-α concentrations were significantly decreased in cocultures containing 5 × 10^4^ cells (*p* < 0.0001) and 2.5 × 10^4^ cells (*p* < 0.01) when compared to cocultures containing 1 × 10^3^ cells/well. These results suggest that cMSCs from synovium, marrow, and adipose tissues are capable of modulating macrophage-mediated inflammation in the described in-vitro coculture system, specifically via modulation of murine TNF-α.

Interestingly, while TNF-α concentrations decreased in response to cMSC coculture, the concentration of murine IL-6 increased in the LPS coculture assay (Additional file 3). The increased concentration of detected IL-6 was not due to cross-reactivity with canine IL-6, because we were unable to detect any IL-6 signal in conditioned media from cMSCs cultured in the absence of murine macrophages (data not shown) and the fact that the selected ELISA is specific for murine IL-6. Cocultures of murine macrophages and canine adipose-derived cMSCs contained significantly higher concentrations of IL-6 when compared to the other tissue types (*p* < 0.001). Collectively, these results indicate that cMSCs are indeed capable of affecting an LPS-mediated inflammatory response in the in-vitro setting, and that when cocultured with cMSCs murine macrophages appear to differentially modulate TNF-α and IL-6.

## Discussion

While the canine species represents a strong model for translation of cell-based treatments from laboratory animals to humans, selecting the ideal tissue source from which to isolate canine MSCs for specific applications remains a challenge. This challenge exists due to the modest number of publications focused on canine MSCs, donor variation across studies, and the widely variable cell isolation, culture, and differentiation protocols used by different groups of investigators. Importantly, authors of existing canine MSC characterization studies for the most part utilized differentiation protocols developed for human MSCs, despite evidence that these protocols in some instances may lead to inconsistent differentiation of canine MSCs [34, 51, 52, 59, 61, 74, 75]. For example, canine MSCs have been shown to be more prone to monolayer detachment in late-stage osteogenic cultures [30, 34, 51, 60, 61, 74], a property we observed during our initial work with canine MSCs. In contrast to human MSCs, in the in-vitro setting, canine MSCs have also been shown to require BMP-2 supplementation for consistent early osteogenic differentiation [52, 75], a finding confirmed in the present study (Fig. 6). With regard to adipogenesis, some authors have described supplementing adipogenic differentiation media with varying concentrations of pantothenate, biotin, rosiglitazone, and rabbit serum to improve the consistency of cMSC adipogenesis [30, 34, 53, 74]. Finally, while the immunomodulatory effects of MSCs have received much attention in other species [77–84, 104–108], only a handful of studies have examined the immunomodulatory potential of canine MSCs [66, 76, 85–88]. As such, immunomodulatory potential of canine MSCs has not typically been included in donor-matched, canine MSC characterization studies involving larger cohorts of dogs.

Synovium, marrow, and adipose tissue were selected for comprehensive characterization because of the strong clinical interest in these tissues, the likely use of cMSCs from these tissues in future translational studies, the ability to acquire these tissues using minimally invasive techniques (bone marrow aspiration and arthroscopy), and the ability of these tissues to produce robust numbers of MSCs in other species. Canine MSCs were successfully isolated from each of the synovium, marrow, and adipose tissue samples obtained from the five canine donors. Canine MSCs were spindle-shaped, adherent to tissue culture plastic, expressed genes associated with pluripotency, and demonstrated colony forming unit (CFU) potential. While these 15 preparations of cMSC met established criteria for human MSCs [37], there were important differences in the number of nucleated cells isolated from the tissues, CFU potential, flow cytometry profile, proliferation rate, trilineage differentiation, and, to a lesser extent, immunomodulatory properties of cMSCs.

Consistent with previous work in humans [28, 109, 110], digests of canine synovium/subsynovial tissue produced large numbers of nucleated cells that exhibited significantly higher CFU potential (6.5%) when compared to marrow (0.01%) or adipose tissue digests (2.6%) (Table 2). Although at present canine synovium has received little attention as a source of MSCs for translational research [111–114], results of the present study demonstrate that canine synovium contains high numbers of MSCs which compare favorably to adipose and marrow-derived cMSCs in the in-vitro setting. In fact, canine synovium exhibited the highest CFU potential of the three examined tissues. Synovium-derived cMSC proliferation was only slightly more attenuated than that of adipose-derived cMSCs and was more rapid than marrow cMSCs. The cells also possessed similar immunomodulatory activity. In differentiation assays, synovium-derived cMSCs performed in a manner similar to marrow-derived cMSCs. These cells underwent robust early-stage and late-stage osteogenesis and adipogenesis, and formed spherical pellets in chondrogenic assays that were larger than marrow-derived pellets and similar to adipose-derived pellets. Toluidine blue staining and collagen type II immunohistochemistry demonstrated reduced proteoglycan and collagen type II staining as compared to marrow-derived pellets but subjectively increased collagen type II staining when compared to adipose-derived cMSC chondrogenic cultures. Collectively, these results demonstrate that canine synovium is an attractive source for harvesting MSCs in future studies.

A relative consensus currently exists regarding the flow cytometry profile for human MSCs [37]. Unfortunately, a consensus regarding an acceptable flow cytometry profile remains to be determined for canine MSCs. However, our basic flow cytometry results are similar to previous cMSC studies, which for the most part describe canine MSCs as consistently CD45^–^, CD9^+^, and CD44^+^ [29, 34, 73, 74, 76, 85, 89, 115, 116]. The canine MSC preparations evaluated in the present study were consistently CD34^–^, CD45^–^, CD9^+^, CD44^+^, and CD90^+^. Our flow cytometry profiling did reveal two interesting findings worthy of discussion. First, the 15 preparations of cMSCs in the present study exhibited extremely low STRO-1 levels. The antigen recognized by the STRO-1 antibody is a component of heat shock cognate 80 (HSC70;HSPA8) [117]. In previous canine MSC literature, a single study defined canine marrow-derived MSCs as positive for STRO-1 [62]. Additionally, Whitworth et al. [70] utilized immunocytochemistry to document STRO-1 in canine iPS-MSCs and primary bone marrow MSCs. While STRO-1 has been linked to colony-forming osteogenic progenitor cells in humans [118, 119], Jhin et al. [120] demonstrated that canine MSCs from periodontal ligament, alveolar bone, and bone marrow were negative for STRO-1. This finding is consistent with Sakaguchi et al. [28], who reported extremely low STRO-1 levels in human synovium-derived MSCs. Explanations for variable STRO-1 staining include the rapid loss of STRO-1 during isolation and culture [121] and/or an inherently variable STRO-1 expression in MSCs isolated from different tissues [31, 38, 122, 123].

Second, CD105 (Endoglin) expression varied dramatically when evaluated based on the tissue of origin of cMSCs. The values for our five canine donors varied from 46.1 ± 21.8% for synovium, 17.1 ± 8.8% for marrow, and 59.8 ± 16.6% for adipose-derived cMSCs (Fig. 2, Additional file 2). CD105 is a high affinity coreceptor for transforming growth factor (TGF)-β1 and TGF-β3 [124]. Canine MSCs have been shown to express CD105 [70, 72, 74, 115]. Although CD105 is considered an important MSC marker linked to trilineage differentiation potential in human cells [37, 125, 126], many studies have demonstrated that CD105 expression levels vary substantially based on the tissue source of MSCs, duration in culture, and differentiation status [127–132]. Regarding the effect of CD105 on trilineage differentiation, it has been shown that increased expression of CD105 in human and rat synovium-derived MSCs promoted chondrogenesis in vitro [127, 133]. In contrast, Cleary et al. [134] reported that the presence of CD105 had no effect on chondrogenesis in human bone marrow-derived MSCs. In murine adipose-derived MSCs, initial stromal vascular fraction preparations were heterogeneous for CD105 and cells negative for CD105 were capable of forming an osteogenic population [124, 131]. Furthermore, expression of CD105 in murine and human adipose-derived MSCs was induced by exposure of cells to tissue culture plastic and was further affected by passage number and confluence [131, 135]. These findings are further supported by additional studies in which CD105-negative MSCs exhibited enhanced adipogenic and osteogenic potential due to reduced TGF-β/SMAD2 signaling [136, 137]. Thus, it appears that in some cases CD105-negative MSCs are indeed capable of undergoing trilineage differentiation. Considering these findings, it is not surprising that the cMSCs isolated from three distinct tissue sources and five unique donors demonstrated variable CD105 staining. It is also possible that, due to the fact that our cMSC preparations were created by plating pooled nucleated cells (no single cell sorting), the cMSC preparations contained a low percentage of contaminating fibroblasts. This may also partially explain the variable CD105 and low STRO-1 results. Future studies are warranted to define the mechanism(s) behind our findings. Furthermore, studies in which cMSCs are characterized for additional flow cytometry markers (i.e. CD73) or sorted by CD105 prior to differentiation and immunomodulation experiments are perhaps of importance for investigators interested in maximizing trilineage differentiation for tissue engineering purposes. Interestingly, it was recently reported that both adipose and marrow-derived cMSCs were negative for CD73 [88].

The ability of MSCs to self-renew and rapidly expand in culture is of considerable importance when selecting a potential tissue source for translational studies. As has been described for human MSCs, the nucleated cell fractions obtained from synovium and adipose tissue digests in the present study exhibited significantly greater CFU potential (Fig. 1) and produced approximately six-fold greater numbers of P0 cMSCs when compared to bone marrow (Table 2). It is important to note that the initial seeding density of the primary nucleated cells varied between synovium/adipose (200 cells/cm^2^) and bone marrow (3 × 10^4^ cells/cm^2^) based on prior literature [28, 138]. Furthermore, the time required to reach 70% confluence for P0 cells varied substantially from 5 to 12 days for all 15 tissue samples, which is consistent with the time required to isolate P0 MSCs in other species. In order to control for both seeding density and the number of days in culture (and thus obtain a more representative head-to-head comparison of the cMSCs), we assessed self-renewal using short-term and long-term proliferation assays (Fig. 4). For all five canine donors, adipose and synovium cMSCs proliferated more rapidly when compared to marrow cMSCs. In our short-term assay, adipose cMSCs proliferated more rapidly than synovium cMSCs, and synovium cMSCs more rapidly than marrow cMSCs, although the latter difference was not statistically significant. Regardless of their source, cMSCs demonstrated substantial proliferation from days 5 to 10, consistent with the lag phase and logarithmic proliferation phases described for human MSCs [28, 96]. In our long-term passaging assay, population doubling of synovium, marrow, and adipose cMSCs was also significantly different. Population doubling of all cell preparations decreased with subsequent passages. Adipose and synovium cMSCs exhibited significantly higher population doubling rates as compared to marrow cMSCs, which further supports our short-term passaging results. Additionally, the long-term passaging assay confirmed the finite capacity of cMSCs to self-renew, a known property of MSCs in other species. When P0 yield and proliferation results are viewed collectively, our findings suggest that while synovium, bone marrow, and adipose tissues each produce cMSCs, investigators requiring rapid expansion of low-passage cMSCs should consider adipose-derived or synovium-derived cMSCs.

An important criterion of MSCs is the ability to differentiate from a progenitor state down multiple mesenchymal lineages. One of the goals of the present study was to utilize optimized differentiation assays relying heavily on previous canine MSC differentiation literature [30, 52, 75] and to evaluate trilineage differentiation of cMSCs using a donor-matched study design and multiple canine donors. The methods and results reported herein will prove useful for investigators unfamiliar with cMSCs, as well as for investigators relying heavily on in-vitro differentiation results to select a source of cMSCs for translational studies.

Adipogenesis was confirmed in all cell preparations using an optimized adipogenic induction protocol, with slight modifications, as defined by Neupane et al. [30]. Importantly, the morphology and size of lipid vacuoles produced after adipogenesis varied based on the tissue source of cMSCs. Adipose and synovium cMSCs produced classic large, grape-like lipid clusters compared to the small, diffuse vacuoles produced by marrow cMSCs (Fig. 5). These morphologic findings were confirmed by semi-quantification of Oil Red O staining. The superior adipogenic differentiation of adipose-derived MSCs is not surprising due to the pericellular cues that are likely provided to adipose cMSCs in their native environment. Interestingly, synovium performed as an intermediate in adipogenic assays, forming large grape-like lipid vacuoles, but forming them with less frequency when compared to adipose tissue. Differences in the expression and/or regulation of critical adipogenic transcription factors peroxisome proliferator activate receptor gamma (PPARγ) and CCAAT enhancer-binding protein (C/EBPα), MEK/ERK signaling, turnover or subcellular localization of B-catenin, or downstream effector proteins of adipocytes such as AcylCoA synthetase (ACS), lipoprotein lipase (LPL), and fatty acid binding protein 4 (FABP4) are likely to explain our adipogenic results [139–141]. Clearly many additional mechanistic studies evaluating the PPARγ signaling axis and other regulators of adipogenesis are necessary to determine the mechanisms behind our findings.

Osteogenic differentiation of cMSCs was assessed using an early kinetic ALP activity assay and the late-stage monolayer ARS mineralization assay. We selected the ALP activity assay because it is a kinetic assay requiring living cells to catabolize an ALP substrate, it has been shown to identify osteogenic differentiation early in the differentiation process [142–144], and it has been evaluated previously in the canine MSC literature. Volk et al. [52] demonstrated that a combination of ascorbate-2-phosphate and rhBMP-2 was necessary to detect ALP activity in early osteogenic marrow-derived cMSC cultures. The results of the present study, in which 15 preparations of cMSCs from three tissue sources were compared, confirm that rhBMP-2 supplementation is necessary to detect ALP activity in early canine osteogenic cultures (Fig. 6). These results demonstrate an important difference between canine and human MSCs which may have in-vivo implications. While none of the cMSCs displayed ALP activity in control (CCM) or basal osteogenic media (OBM) at 7 days of culture, synovium and marrow cMSCs exhibited a strong response to rhBMP-2 as compared to adipose-derived MSCs, with marrow exhibiting the highest ALP activity within each donor. Interestingly, the failure of adipose-derived cMSC to respond to BMP-2 supplementation in ALP activity assays described in the present study confirm the findings of Levi et al. [59], in which treatment of canine adipose stromal cells with osteogenic medium supplemented with 200 ng/ml rhBMP-2 did not increase ALP quantification over baseline. These findings are not surprising given the source of adipose MSCs, and that signaling pathways such as PPARγ compete with osteogenic differentiation pathways such as the canonical Wnt pathway [145–149]. Two potential explanations for the importance of BMP-2 supplementation in canine osteogenesis assays include either species-specific differences in bone biology or the loss of canine BMP-2 expression after cMSC isolation, leading to an absence of endogenous canine BMP-2 during in-vitro osteogenesis. In support of these potential explanations, pilot studies in our laboratory revealed that canine BMP-2 is transcribed at an extremely low level under control or traditional osteogenic conditions in early cMSC osteogenic cultures, requiring greater than 35 cycles to reach detection levels using qPCR (unpublished observations). In contrast, human MSCs robustly transcribe BMP-2 under similar conditions [150–153]. In-vivo evidence supporting the relevance of our in-vitro findings also exists. Canine and murine adipose-derived stromal cells seeded on a hydroxyapatite/PLLA scaffold failed to stimulate defect healing in a murine calvarial defect model, whereas human adipose-derived cells initiated significant healing as early as 2 weeks post treatment [59]. In this regard, it is possible that the differentiation pathways underlying canine adipose-derived MSC osteogenic differentiation as well as the ability of adipose cMSCs to contribute to massive bone loss in xenogenic models may more closely resemble murine as opposed to human MSCs.

Using the modified late-stage osteogenesis methods described herein (mechanical scoring of tissue culture surface, precoating with fibronectin, and induction of osteogenesis with a medium containing rhBMP-2), all three tissues produced calcium-binding mineral (Fig. 7). Consistent with the ALP activity results and previous findings of Volk et al. [52, 75], rhBMP-2 supplementation was required for monolayer mineralization. Adipose and synovium cMSCs demonstrated higher ARS recovery values in some donors, although these differences were not significant when evaluated collectively across all donors. Thus, while adipose cMSCs appear to be resistant to early osteogenic differentiation even in the presence of rhBMP-2, our late-stage osteogenesis results suggest that adipose-derived cMSCs are capable of transitioning to osteogenic cultures over time. As such, investigators considering adipose tissue as a source for cMSC osteogenic cells should consider supplementing osteogenic induction media with rhBMP-2 and realize that additional time in culture may be necessary to achieve osteogenic differentiation.

The osteogenic results for synovium cMSCs in our assays were consistent with prior synovium MSC literature [28, 154]. Human synovium-derived MSCs have been shown to exhibit robust ARS stain in late-stage cultures [28], although to our knowledge early osteogenic differentiation of synovium MSCs has not been examined previously using the ALP activity assay in any species. Given the fact that synovium is a robust source of cMSCs, that synovium demonstrates high CFU potential, and that synovium cMSCs undergo both early and late-stage osteogenesis, synovium may be considered a strong alternative to bone-marrow-derived cMSCs for investigators requiring rapid production of large numbers of cMSCs with early osteogenic potential. Additionally, we have demonstrated that synovium cMSCs exhibit robust ALP activity in early-stage cultures when treated with osteogenic medium containing rhBMP-2.

In contrast to the adipogenic and osteogenic differentiation assays, the serum-free micromass chondrogenesis technique did not require major modifications for use with cMSCs. All 15 cMSC preparations underwent condensation in response to chondrogenic medium and adopted a spherical, translucent appearance classically associated with chondrogenic differentiation. When evaluated histologically, these pellets exhibited both toluidine blue and collagen type II staining, although variability existed based on the tissue source of cMSCs (Fig. 8). Synovium and adipose cMSC produced larger pellets as compared to marrow, but demonstrated subjectively reduced staining for proteoglycan (toluidine blue) and collagen type II when assessed histologically. Despite their small size, marrow-derived chondrogenic pellets were the only cell preparations to consistently exhibit intense staining for proteoglycan (toluidine blue) and collagen type II (immunohistochemistry); however, one obvious limitation regarding the chondrogenic capacity of marrow cMSCs is the reduced pellet size when compared to synovium and adipose cMSCs. Synovium cMSCs provided intermediate chondrogenic results with pellets considerably larger than those produced by marrow cMSCs but containing slightly reduced proteoglycan and collagen type II staining. Adipose cMSCs produced large structures; however, these structures subjectively contained the lowest proteoglycan and collagen type II staining. Admittedly, additional metrics to compare chondrogenic differentiation across donors and tissue types are needed. Future studies in our laboratory that focus solely on chondrogenic differentiation of cMSCs will not only rely on traditional histology and morphometry, but will also utilize quantitative assessment of proteoglycan content and transcriptional activity. The goal of the present study was to provide broad characterization using a donor-matched study design in order to describe general similarities and differences in canine MSCs. Additional quantitative assessments of chondrogenesis for 15 preparations of cMSCs was beyond the scope of the study. Furthermore, additional work is necessary to optimize cMSC chondrogenesis to produce larger micromass pellets with high-intensity staining for proteoglycans and collagen type II for canine translational cartilage repair studies.

 In the current MSC field, in order to be considered an MSC, a cell must exhibit immunomodulatory potential. While the immunomodulatory potential of human MSCs is well described, a handful of studies have reported that canine marrow, adipose, and periodontal ligament-derived MSCs are capable of producing growth factors, producing anti-inflammatory cytokines, or directly modulating leukocyte activity or proliferation [66, 76, 85–88]. However, the immunomodulatory potential of synovium-derived cMSCs has yet to be examined and immunomodulatory assays have not been included in large donor-matched canine MSC characterization studies. The first description of immunomodulatory potential of cMSCs was provided by Kang et al. [85]. Adipose-derived cMSCs were isolated from a single donor and examined using a comprehensive series of immunomodulatory assays. Adipose cMSCs expressed baseline mRNA for a number of anti-inflammatory proteins, cytokines, and growth factors. The inflammatory cytokine TNF-α decreased in leukocyte and cMSC cocultures, whereas the immunomodulatory agents TGF-β, hepatocyte growth factor (HGF), prostaglandin E2 (PEG2), and indoleamine 2,3 dioxygenase (IDO) increased. Furthermore, proliferation of stimulated leukocytes was suppressed when cocultured with irradiated cMSCs or with cMSC conditioned media. In support of Kang’s findings, Park et al. [87] demonstrated that adipose-derived cMSCs inhibited T-cell proliferation after in-vivo MSC transplantation. With regard to marrow-derived cMSCs, Lee et al. [66] reported that canine marrow MSCs were capable of inhibiting leukocyte proliferation and implicated cMSC-derived PGE2 as a potential anti-proliferative factor. Interestingly, while marrow cMSCs displayed in-vitro characteristics similar to MSCs from other species, cMSCs failed to sustain bone marrow engraftment in vivo using a total body irradiation and dog leukocyte antigen (DLA) matched experimental design. In the most comprehensive in-vitro canine immunomodulatory study to date, Chow et al. [88] compared the immunomodulatory properties of adipose and marrow cMSCs from three unrelated dogs. While adipose and marrow cMSCs were roughly equivalent in their ability to suppress T-cell activation, the cMSCs utilized both shared and distinct pathways to accomplish T-cell suppression. An elegant series of coculture experiments and microarrays was used to detail the differences and similarities between adipose and marrow cMSC immunomodulatory potential.

Given the previous studies already detailed, the authors suggest that immunomodulatory assays should be included in characterization assays aimed at confirming canine MSC identity. Moreover, we propose that assessment of immunomodulatory potential should be considered an important metric in comparative characterization studies involving multiple preparations of cMSCs derived from diverse tissues. As such, we aimed to determine whether cMSCs had the capacity to inhibit specific features of the innate immune response and to determine whether the tissue of origin affected this process using a macrophage coculture assay. When murine macrophages were treated with LPS, there was a robust secretion of murine TNF-α that could be detected by ELISA. Inclusion of cMSCs resulted in a significant and dose-dependent decrease in the measured concentration of secreted murine TNF-α. Furthermore, this response was observed for all 15 cMSC cell preparations, while the tissue source of cMSCs did not affect the reduction of measured TNF-α. The reduction in TNF-α in the present report is consistent with previous adipose-derived cMSC work [85]. To the authors’ knowledge, the present study is the first report to confirm that canine synovium-derived cMSCs exhibit immunomodulatory potential.

It has been described previously that toll-like receptor (TLR) activation is critical for LPS-mediated immunomodulation [155]. However, recent studies describe a noncanonical signaling pathway in which an immune response is elicited without LPS–TLR4 binding [156, 157]. These canonical and noncanonical regulatory mechanisms may be partially responsible for our IL-6 results, namely that inclusion of cMSCs in LPS-stimulated cocultures resulted in an increased concentration of murine IL-6 in coculture conditioned medium. One explanation for increased production of murine IL-6 in response to cMSCs exposed to inflammatory stimuli is the initiation of a proinflammatory pathogen clearance mechanism [158]. This may occur through the NF-κB pathway and PGE production [103, 155]. Additional experiments beyond the scope of the present study are needed to determine the exact regulatory pathways involved in this process.

Despite numerous advantages, limitations of the canine model must also be considered. While many clinically impactful studies have been completed using the canine model, the dog is not traditionally considered to be a common large animal research species. This is particularly the case in societies in which dogs are often considered at a minimum as in-home pets and by some as family members. However, improvements in research protocols to meet changing ethical standards and the adoption of a “one-heath” approach to many medical problems have led to altered perceptions regarding the importance and impact of canine translational studies. For these reasons, the authors suggest that investigators interested in canine clinical models consider collaborating with small animal veterinary clinician scientists or research teams with extensive canine experience. The numerous advantages of these types of collaborative approaches were reviewed recently [25]. While the canine genome has been successfully sequenced, the number of mRNA transcripts that have been sequenced and confirmed as identical to predicted mRNA transcripts is much more modest than in other model species. This may lead to challenges in designing effective PCR primers or siRNAs. Additionally, there is a smaller pool of commercially available reagents capable of cross-reacting with the canine species. For example, sourcing antibodies for flow cytometry, immunohistochemistry, and western blotting applications can be a challenge in some instances. Lastly, while the human and dog species share many similarities with regard to skeletal biomechanics, hematopoietic function, and cardiovascular physiology, important differences do exist between these species. The need to supplement canine MSCs with rhBMP-2 for successful early-stage osteogenesis reported in the present study is one such example. Thus, caution should be taken when making assumptions between species or when attempting to translate research findings from one species directly to the other without confirming studies. While in-vitro MSC characterization studies represent a critically important foundation for discovery, differences in cMSC performance in vitro may or may not be relevant to specific disease states in vivo (either spontaneous or induced injury). As with all in-vitro work, the clinical impact of the present study should be confirmed with future studies in which in-vitro cMSC characterization can be directly compared alongside outcomes in vivo.

## Conclusions

We successfully isolated MSCs from canine synovium, marrow, and adipose tissues. While all cMSC preparations exhibited characteristics of MSCs using in-vitro assays optimized for the canine species, both the tissue of origin and the donor impacted cMSC performance. Synovium cMSCs exhibited robust early-stage and late-stage osteogenic differentiation. Combining their ease of isolation, CFU potential, rapid proliferation, immunomodulatory potential, and presence within the intra-articular niche, canine synovium MSCs appear to be an excellent choice for orthopedic translational cell-based studies. While marrow cMSCs had a lower CFU potential and proliferated more slowly, our findings demonstrate that marrow cMSCs were capable of marked early and late-stage osteogenic differentiation and produced chondrogenic pellets that stained intensely for proteoglycan and collagen type II, making marrow an excellent source of cMSCs for orthopedic applications. Given the inability of adipose cMSCs to demonstrate detectible ALP activity even in the presence of substantial BMP-2 supplementation, adipose tissue may not be an ideal source for osteogenic cells if short-term cultures are required; however, adipose tissue produced large numbers of cMSCs with high CFU and proliferation potential. Moreover, adipose cMSCs produced calcium-rich late-stage monolayer osteogenic cultures, and thus may be suitable for investigators interested in long-term culture of tissue engineering constructs. Interestingly, cMSCs isolated from synovium, marrow, and adipose tissue modulated TNF-α levels in LPS-stimulated macrophage coculture assays, suggesting that the three tissue sources of cMSCs are capable of immunomodulatory activity in the in-vitro setting. Our methods and results provide insight into important similarities and differences between cMSCs and human MSCs, and will prove useful for investigators considering these canine tissues for large animal translational studies.

## Additional files

Additional file 1: Supplemental Materials & Methods containing a complete/comprehensive description of the materials and methods, which are summarized in the primary manuscript. (DOCX 60 kb)

Additional file 2: Table S1. Presenting flow cytometry results. (DOCX 17 kb)

Additional file 3: Figure S1. Showing immunomodulation of murine IL-6. (TIFF 427 kb)

## Abbreviations

ALP: Alkaline phosphatase
ANOVA: Analysis of variance
ARS: Alizarin red stain
AUP: Animal use protocol
BMP-2: Bone morphogenic protein-2
CCM: Complete culture medium
cDNA: Complementary DNA
CFU: Colony forming unit
cMSC: Canine MSC
ELISA: Enzyme-linked immunosorbent assay
FBS: Fetal bovine serum
IACUC: Institutional animal care and use committee
IL-6: Interleukin 6
MSC: Multipotent stromal cell
OBM: Osteogenic basal medium
ODM: Osteogenic differentiation medium
PBS: Phosphate-buffered saline
PD: Population doubling
P-NPP: *p*-Nitrophenyl phosphate
SD: Standard deviation
TLR: Toll-like receptor
TNF-α: Tumor necrosis factor alpha
