# Supplementary material for: In-vitro characterization of canine multipotent stromal cells isolated from synovium, bone marrow, and adipose tissue: a donor-matched comparative study
Source: Stem Cell Res Ther. 2017 Oct 3;8:218. doi: 10.1186/s13287-017-0639-6 (PMC5627404; doi:10.1186/s13287-017-0639-6)
Supplement: Supplementary file 1 — Supplemental Materials & Methods containing a complete/comprehensive description of the materials and methods, which are summarized in the primary manuscript. (DOCX 60 kb) [file 13287_2017_639_MOESM1_ESM.docx]

**In Vitro Characterization of Canine Multipotent Stromal Cells Isolated from Synovium, Bone Marrow, and Adipose Tissue: A Donor-Matched Comparative Study**

**Names of Authors:**

Robert N. Bearden- lead author

Department of Small Animal Clinical Sciences, College of Veterinary Medicine and Biomedical Sciences, Texas A&M University, College Station, TX.

Email: rbearden@cvm.tamu.edu

Shannon S. Huggins

Department of Small Animal Clinical Sciences, College of Veterinary Medicine and Biomedical Sciences, Texas A&M University, College Station, TX.

Email: shuggins@cvm.tamu.edu

Kevin J. Cummings

Department of Veterinary Integrative Biosciences, College of Veterinary Medicine and Biomedical Sciences, Texas A&M University, College Station, TX.

Email: kcummings@cvm.tamu.edu

Roger Smith

Department of Veterinary Pathobiology, College of Veterinary Medicine and Biomedical Sciences, Texas A&M University, College Station, TX.

Email: rosmith@cvm.tamu.edu

Carl A. Gregory

Department of Molecular and Cellular Medicine, Institute for Regenerative Medicine, College of Medicine, Texas A&M University, College Station, TX.

Email: cgregory@medicine.tamhsc.edu

William B. Saunders- corresponding author

Department of Small Animal Clinical Sciences, College of Veterinary Medicine and Biomedical Sciences, Texas A&M University, College Station, TX.

Email: bsaunders@cvm.tamu.edu

**Additional File 1: Supplemental Materials & Methods**

**Tissue Collection and Cell Isolation:**

This study was performed under the supervision of the institutional animal care and use committee (IACUC) and an approved animal use protocol (AUP 2011-149). Canine synovium, bone marrow, and adipose tissues were obtained from four-castrated male and one spayed female dogs during knee arthroscopy for cranial cruciate ligament rupture (Table 1). Median age was three years (range 2 to 6) with a median body weight of 39.4 kg (range 21.4 to 71.7 kg). Under general anesthesia, bone marrow aspirates were performed on the proximal humerus using 15 gauge Illinois biopsy needles. Adipose tissue was obtained from the infrapatellar fat pad prior to arthroscope insertion. Synovium/subsynovial tissues were isolated from the femoropatellar joint using arthroscopic biopsy forceps during knee arthroscopy. Sample weights and volumes are provided in Table 1.

Nucleated cells were isolated from bone marrow using gradient centrifugation (Ficoll-Paque Plus, GE Health Care Biosciences, Piscataway, NJ) as previously described

[1]. Following centrifugation at 1,800xG for 30 minutes, mononuclear cells were removed, washed twice with 15mL of Hank’s Balanced Salt Solution (HBSS, Invitrogen, Carlsbad, CA), quantified, and assessed for viability using a hemocytometer and trypan blue exclusion. Adipose and synovial samples were washed in HBSS and were manually minced into a paste consistency using sterile scalpel blades and scissors. Samples were subjected to enzymatic digestion using Liberase TM® (Roche Molecular Biochemical, Mannheim, Germany) with gentle agitation at 37**°**C for 3-6 hours[2]. Digested samples were washed, centrifuged, and strained though a 40µm cell strainer. Residual non-digested tissues that were retained in the cell strainer were discarded. Strained cells were washed and centrifuged for quantification and assessment of viability.

Nucleated marrow cells were plated at 3x10^4^ cells/cm^2^ in 150cm^2^ tissue culture dishes in Complete Culture Medium (CCM) consisting of αMEM, 100U/mL penicillin, 100µg/mL streptomycin (Invitrogen), and 10% premium select fetal bovine serum (Atlanta Biological, Inc., Flowery Branch, GA), while nucleated cells from adipose and synovium were plated at 200 cells/cm^2^. Cells were incubated at 37**°**C and 5% humidified CO_2_ for 24 hours. For the following three days, plates were washed with PBS to remove non-adherent cells followed by media exchange. Culture dishes were subsequently monitored for expansion of the primary cell population (passage 0) with media exchange performed every other day. At 70% confluence (5-12 days) cells were lifted with 0.5% trypsin/EDTA solution (Invitrogen) and re-seeded at 100 cells/cm^2^ for expansion of passage 1 cells. Media was exchanged every other day until cells were 70% confluent. Passage 1 cells were cryopreserved in αMEM with 5% DMSO (Sigma-Aldrich, St. Louis, MO) and 30% FBS in preparation for subsequent experiments. With exception of the colony forming unit (CFU) assays, passage 1 cells were thawed, plated at 100 cells/cm^2^, and expanded to 70% confluent passage 2 cells for use in experiments.

**Colony Forming Unit (CFU) Assay:**

CFU ability of the primary cell population was determined on the day of tissue harvest by plating isolated cells in triplicate on 55cm^2^ dishes at 4.5x10^5^ total cells/dish for bone marrow and 1x10^3^ total cells/dish for synovium and adipose tissue as previously described

[3-5]. Cells were incubated at 37**°**C in 5% humidified CO_2_ for 24 hours in CCM. Plates were washed with PBS twice followed by media exchange with CCM at 24 and 48 hours. After the 48-hour wash, plates were incubated 21 additional days without media exchange. At 21 days, plates were stained with 0.3% crystal violet solution (Sigma-Aldrich) for 30 minutes. Plates were washed, photographed, and colony number and size were quantified using Image J FIJI Colony Counter[6].

**RT-PCR for Pluripotency-Associated Genes:**

Isolation of RNA and subsequent gene expression evaluation was performed as previously described[7]. Total RNA was isolated from passage 2 cells using PureLink™ RNA Mini Kit (Life Technologies, Carlsbad, CA) and treated with DNase to remove contaminating DNA. Complementary DNA (cDNA) was synthesized from 2.5µg of total RNA using random hexamer primers and Superscript III reverse transcriptase (Invitrogen). Primers were commercially synthesized (Sigma-Aldrich) as follows: GAPDH[8] Forward: GGAGAAAGCTGCCAAATATG, Reverse: ACCAGGAAATGAGCTTGACA; NANOG[7] Forward: GAATAACCCGAATTGGAGCAG, Reverse: AGCGATTCCTCTTCACAGTTG; OCT4[7] Forward: GAGTGAGAGGCAACCTGGAG, Reverse: GTFAAGTGAGGGCTCCCATA; SOX2[7] Forward: AGTCTCCAAGCGACGAAAAA, Reverse: GCAAGAAGCCTCTCCTTGAA. PCR reactions (20µL) were prepared with 2µL of cDNA, 10nM of each primer, and 0.5 units of AmpliTaq Gold**®** 360 DNA Polymerase (Invitrogen). Cycling conditions were performed with an initial denaturation at 94**°**C for 5 minutes, followed by 35 cycles of: denature at 94**°**C for 0.5 minutes, anneal at 55**°**C for 0.5 minutes, extend at 72**°**C for 0.5 minutes, and a final extension at 72**°**C for 7 minutes. Upon completion, PCR products were separated on a 1.2% agarose gel by constant voltage electrophoresis for 60 minutes. PCR products were visualized using Gel Green (Biotium, Hayward, CA) and images captured using BioRad Chemdoc™MP Imager and ImageLab software (version 5.2.1; Biorad, Hercules, CA).

**Flow Cytometry:**

Passage 2 cMSCs were trypsinized, washed in PBS, counted, resuspended to 6x10^5^ cells/mL, and divided into individual aliquots containing 3x10^5^ cells in 50µL of PBS and 2% FBS. Commercially available antibodies were acquired for cell analysis from AbD Serotec (CD-9, -34, -44, -45, -90; Raleigh, North Carolina), Santa Cruz (CD-105; Santa Cruz, CA), and R&D (STRO-1; Minneapolis, MN). Cells were incubated in primary antibody (CD34-, 44-, 45-, 90- 100µg/mL, CD9- 10µg/mL, CD105- 40µg/mL, STRO1- 150µg/mL) for 30 minutes, washed twice, and resuspended in 300µL of PBS and 2% FBS. For CD44, CD90, and STRO-1 cells were resuspended in 50µL of PBS and 2% FBS for incubation in secondary antibody at 6.25µg/mL for 30 minutes, then washed twice, and resuspended in 300µL of PBS and 2% FBS for analysis

[9,10]. Cell fluorescence was determined by flow cytometry using a FACSCalibur flow cytometer (BD Biosciences, San Jose, CA), CellQuest (BD Biosciences) acquisition software, and FlowJo analysis software version 9.8.3 (TreeStar, Inc, Ashland, Oregon). Prior to analysis, propidium iodide was added at 100µg/mL to identify and remove non-viable cells. The filters used were 610/20 for propidium iodide and 530/30 for FITC.

**Proliferation Assays:**

**Short-term Proliferation:**

To compare the short-term proliferation of synovium, marrow, and adipose cMSCs, cells were plated at 100 cells/cm^2^ in triplicate wells on 12-well tissue culture plates in CCM as described above. Cells were washed with PBS, fixed in 500µL of DNA quantification buffer at 24-hour intervals for 10 days, and quantified by fluorescence DNA incorporation assay as previously described[11]. After application of DNA quantification buffer, cells were frozen and stored at -20**°**C until assay completion. After thawing to room temperature, cell monolayers were digested by adding 1mg/mL collagenase (Sigma), 1U/mL HINDIII (Invitrogen), and 1U/mL ECOR (Invitrogen) for incubation at 37**°**C for 16 hours. The following day, cell suspensions were transferred to 1.5mL tubes and Sytox Orange (Life Technologies) was added in a 1000-fold dilution. Cell suspensions were centrifuged at 15,000xG for 20 minutes and supernatant added to opaque 96-well plates in triplicate to determine fluorescence[11]. Individual cell numbers were determined by referencing a linear standard curve consisting of pre-determined numbers of cMSCs.

**Long-term Proliferation:**

To compare the long-term proliferation of cMSCs over multiple passages, cells were plated in triplicate at 100 cells/cm^2^ on 55cm^2^ dishes and cultured in CCM with media exchange every other day. After five days, cells were trypsinized, manually counted, and re-plated at 100 cells/cm^2^. This process was repeated for a total of five cell passages (25 cumulative days in culture). At each passage, cell yield per plate was determined using a hemocytometer and trypan blue exclusion (n=3 plates/cell line) and data reported as the mean population doubling number for each cell preparation. Population doubling was determined as previously described by Greenwood et al. as follows: population doubling (PD) is equal to the log of the ratio of the final cell count (N) to the starting cell count (X_0_), divided by the log of 2; that is PD= [log(N ÷ X_0_)] ÷ log2[12].

**Adipogenesis:**

Canine MSCs were plated in CCM at 2x10^4^ cells/cm^2^ in 12-well plates (n=4 wells/condition). The following day, cells were treated with control medium (CCM) or modified adipogenic medium developed based on slight modifications to prior canine adipogenic differentiation publications

[2,7,13-17]. Adipogenic medium consisted of αMEM containing 1nM dexamethasone (Sigma), 5mM rosiglitazone (Sigma), 50mM pantothenate (Enzo Life Sciences, Farmingdale, NY), 10mM insulin (Sigma), 30mM biotin (Enzo), 50mM isobutylmethylxanthine (Sigma), and 10% serum [5% FBS, 5% rabbit serum (Atlanta Biological)]. Media were exchanged twice weekly. After 21 days, cells were washed with PBS and fixed in 10% neutral buffered formalin prior to staining in 0.5% Oil Red O (Sigma) to document lipid vacuole formation. Cells were photographed prior to extraction of Oil Red O stain for quantification as previously described[2].

**Early Assay of Osteogenesis - Alkaline Phosphatase (ALP) Activity:**

Canine MSCs were plated in CCM at 5x10^3^ cells/cm^2^ in 12-well plates (n=3 wells/condition). The following day (Day 1), cells were treated with control medium (CCM), or osteogenic basal medium optimized for the dog. Osteogenic basal medium (OBM) consisted of αMEM, 5% FBS, 10µg/mL beta-glycerophosphate (BGP, Sigma), 50mg/mL ascorbate-2-phosphate (Sigma), or OBM supplemented with 50 or 100-ng/mL of recombinant human bone morphogenic protein-2 (rhBMP-2; R&D Systems) as previously described[11,18]. Media were exchanged on day four. At day seven, cells were washed twice with PBS and incubated with 500µL of 4°C ALP activity buffer containing 1mM magnesium chloride (Sigma), 0.1% Triton-X (Sigma), and 100mM sodium chloride (Sigma) in PBS. 500µL of 4°C ALP substrate p-nitrophenylphosphate (P-NPP, Thermo Fisher Scientific; West Palm Beach, FL) was added to each well to initiate the assay. Absorbance was determined for each well at 405nm in one minute intervals for 20 minutes at 37°C using an automated plate reader (HT Synergy, BioTek, Winooski, VT) and Gen3Bio software. Kinetic ALP activity curves were generated for each well and the ALP activity per well was calculated by determining the slope of each activity curve using a linear curve fit technique. ALP activity of each well was normalized to the number of cells in each well using the DNA quantification technique described above[11].

**Late-stage Assay of Osteogenesis - Alizarin Red Stain (ARS) Mineralization:**

Detachment of high-density monolayers from polystyrene tissue culture plastic in late-stage mineralization assays is a phenomenon that is not uncommon in cMSC mineralization assays. We identified this phenomena in our early workings with cMSCs (unpublished observations) and this problem has been described in prior cMSC literature[18-20]. In order to prevent monolayer detachment, cMSCs were plated in CCM at 2x10^4^ cells/cm^2^ in 12-well plates (n=4 wells/condition). Prior to plating, the periphery of each well was mechanically scored using a sterile stone dremel bit and associated hand chuck in order to create a circular etching around the well margin. Wells were coated with human fibroblast derived fibronectin (Sigma) at 5mg/mL in PBS for 30 minutes at 37°C. Excess fibronectin was removed and cells were seeded in each well. The following day, cells were treated with CCM, OBM, or OBM supplemented with 200ng/mL rhBMP-2. Media were exchanged twice weekly. After seven days, 1nM dexamethasone was added to OBM and OBM + BMP-2 wells, creating osteogenic differentiation media (ODM) to induce mineralization

[11,17,18]. At 21 days, cells were washed with PBS and fixed in 500µL of 10% neutral buffered formalin prior to staining in 40nM alizarin red stain (ARS; Sigma) to visualize calcium deposition within osteogenic monolayers. Wells were photographed prior to extraction of ARS for semi-quantification via spectrophotometry using an acetic acid extraction technique as previously described[11].

**Chondrogenesis:**

Micromass cultures of cMSCs were generated from 5x10^5^ cells using previously described techniques[2]. Briefly, passage 2 cMSCs were washed in PBS, trypsinized, counted, and resupsended to 1x10^6^ cells/mL in chondrogenic medium. Chondrogenic medium consisted of DMEM supplemented with a 1:250 dilution of ITS+ culture supplement, 50µg/mL ascorbate-2-phosphate, 40µg/mL L-proline (Sigma), 100µg/mL sodium pyruvate (Sigma), 10^-7^M dexamethasone, 10ng/mL recombinant human transforming growth factor-β3 (rhTGF-β3, R&D Systems), and 500ng/mL of rhBMP-2. Cells were pelleted in 15mL conical tubes (5x10^5^ cells/tube) by centrifugation at 500xG for 5 minutes (n=3 pellets/cell line). Chondrogenic medium was exchanged twice weekly. After 21 days, chondrogenic pellets were fixed in 10% neutral buffered formalin. Pellets were photographed and area (mm^2^) was determined using digital morphometry. Pellets were subsequently embedded in paraffin and sectioned for histologic evaluation using 1% toluidine blue/sodium borate (Sigma) stain.

**Immunohistochemistry:**

Paraffin-embedded sections were deparaffinized in CitraSolv (Thermo Fisher Scientific) and rehydrated using a graded alcohol series. Antigen retrieval was performed with boiling citrate buffer and endogenous peroxidase activity was quenched by incubating sections in methanol containing 1% hydrogen peroxide for 15 minutes at room temperature. Sections were then incubated with rabbit anti-collagen type II IgG (Abcam, Cambridge, MA; 10μg/ml in PBS containing 1% BSA) or rabbit IgG (Rockland, Limerick, PA) as a negative control for 16 hours at 4°C. Immunoreactive protein was visualized using the Vectastain ABC Kit (Vector Laboratories Inc., Burlingame, CA) according to kit instructions with 3,3′-diaminobenzidine tetrahydrochloride (Sigma) as the color substrate. Sections were lightly counterstained with Gill's hematoxylin, dehydrated, and coverslipped for photography[21,22].

**Immunomodulation:**

To assess macrophage-mediated immunomodulation, mouse macrophage cells (RAW 264.7 cell line, American Type Culture Collection TIB-71) were seeded at 1x10^4^ cells/cm^2^ in 12-well plates in CCM and allowed to attach overnight. After 24 hours (approximately 60% confluence), cMSCs were titrated (1x10^3^, 1x10^4^, 2.5x10^4^, and 5x10^4^ cells/well) to initiate a 24 hour co-culture (n=3 replicates/cMSC dosage). Lipopolysaccharide (*E. coli* 055:B5 strain, Sigma) was introduced to each well at 0.5µg/mL to induce macrophage activation. Co-cultures were allowed to respond for 18 hours, at which point conditioned media were collected and stored at -20°C. Upon evaluation of all 15 cMSC preparations, media were thawed on ice and analyzed for murine TNF-α (Product Code DY410-05) and IL-6 (Product Code DY406-05) protein concentrations via enzyme-linked immunosorbent assay (ELISA) according to manufacturer’s protocol (R&D).

**Statistical Analysis:**

Descriptive statistics were generated using GraphPad Prism 6.0 (GraphPad Software, La Jolla, CA) and were reported as mean ± standard deviation. Data were imported into a commercial statistical software program (SAS, version 9.4; SAS Institute Inc., Cary, NC, USA) for inferential statistics. Repeated measures ANOVA was used to determine if each parameter differed significantly by tissue type and treatment group, as appropriate, with donor dog regarded as a random effect. The Tukey method was used to adjust for multiple pairwise comparisons. For all analyses, p-values < 0.05 were considered significant.

**References**

1. Pittenger MF. Mesenchymal stem cells from adult bone marrow. Methods Mol. Biol. 2008;449:27–44.

2. Sekiya I, Larson BL, Smith JR, Pochampally R, Cui JG, Prockop DJ. Expansion of human adult stem cells from bone marrow stroma: Conditions that maximize the yields of early progenitors and evaluate their quality. Stem Cells. 2002;20:530–41.

3. Pochampally R. Colony forming unit assays for MSCs. Methods Mol. Biol. 2008;449:83–91.

4. Prockop D, Sekiya I, Colter D. Isolation and characterization of rapidly self- renewing stem cells from cultures of human marrow stromal cells. Cytotherapy. Elsevier; 2001;3:393–6.

5. Digirolamo C, Stokes D, Colter D, Phinney D, Class R, Prockop D. Propagation and senescence of human marrow stromal cells in culture. British Journal of Haematology. 1999;107:275–81.

6. Schneider C, Rasband W, Eliceiri K. NIH image to ImageJ: 25 years of image analysis. Nature Methods. Nature Publishing Group; 2012;9:671–5.

7. Neupane M, Chang C, Kiupel M, Yuzbasiyan-Gurkan V. Isolation and characterization of canine adipose–derived mesenchymal stem cells. Tissue Engineering Part A. 2008;14:1007–15.

8. Okui Y, Kano R, Maruyama H, Hasegawa A. Cloning of canine Toll-Like receptor 7 gene and its expression in dog tissues. Veterinary Immunology and Immunopathology. 2008;121:156–60.

9. Stewart K, Walsh S, Screen J, Jefferiss C, Chainey J, Jordan G, et al. Further characterization of cells expressing STRO-1 in cultures of adult human bone marrow stromal cells. Journal of Bone and Mineral Research. 1999;14:1345–56.

10. Stewart C, Stewart J. Cell preparation for the identification of leukocytes. Methods in Cell Biology. 2001;63:217–51.

11. Krause U, Seckinger A, Gregory CA. Assays of osteogenic differentiation by cultured human mesenchymal stem cells. Methods in Molecular Biology. Totowa, NJ: Humana Press; 2011. pp. 215–30.

12. Greenwood S, Hill R, Sun J, Armstrong M, Johnson T, Gara J, et al. Population doubling: A simple and more accurate estimation of cell growth suppression in the in vitro assay for chromosomal aberrations that reduces irrelevant positive results. Environ. Mol. Mutagen. 2004;43:36–44.

13. Schwarz C, Leicht U, Rothe C, Drosse I, Luibl V, Röcken M, et al. Effects of different media on proliferation and differentiation capacity of canine, equine and porcine adipose derived stem cells. Research in Veterinary Science. Elsevier Ltd; 2012;93:457–62.

14. Al-Nbaheen M, Vishnubalaji R, Ali D, Bouslimi A, Al-Jassir F, Megges M, et al. Human stromal (mesenchymal) stem cells from bone marrow, adipose tissue and skin exhibit differences in molecular phenotype and differentiation potential. Stem Cell Rev and Rep. 2012;9:32–43.

15. Csaki C, Matis U, Mobasheri A, Ye H, Shakibaei M. Chondrogenesis, osteogenesis and adipogenesis of canine mesenchymal stem cells: A biochemical, morphological and ultrastructural study. Histochem Cell Biol. 2007;128:507–20.

16. Kisiel A, McDuffee L, Masaoud E, Bailey T, Esparza Gonzalez B, Nino-Fong R. Isolation, characterization, and in vitro proliferation of canine mesenchymal stem cells derived from bone marrow, adipose tissue, muscle, and periosteum. American Journal of Veterinary Research. 2012;73:1305–17.

17. Reger RL, Tucker AH, Wolfe MR. Differentiation and characterization of human MSCs. Methods Mol. Biol. 2008;449:93–107.

18. Volk S, Diefenderfer D, Christopher S, Haskins M, Leboy P. Effects of osteogenic inducers on cultures of canine mesenchymal stem cells. American Journal of Veterinary Research. 2005;66:1729–37.

19. Volk S, Wang Y, Hankenson K. Effects of donor characteristics and ex vivo expansion on canine mesenchymal stem cell properties: Implications for MSC-based therapies. cell transplant. 2012;21:2189–200.

20. Kadiyala S, Young R, Thiede M, Bruder S. Culture expanded canine mesenchymal stem cells possess osteochondrogenic potential in vivo and in vitro. cell transplant. 1997;6:125–34.

21. Spencer T, Bartol F, Bazer F, Johnson G, Joyce M. Identification and characterization of glycosylation-dependent cell adhesion molecule 1-like protein expression in the ovine uterus. Biology of Reproduction. 1999;60:241–50.

22. Gao H, Wu G, Spencer T, Johnson G, Bazer F. Select nutrients in the ovine uterine lumen. II. Glucose transporters in the uterus and peri-implantation conceptuses. Biology of Reproduction. 2008;80:94–104.
