# Supplementary material for: In-vitro characterization of canine multipotent stromal cells isolated from synovium, bone marrow, and adipose tissue: a donor-matched comparative study
Source: Stem Cell Res Ther. 2017 Oct 3;8:218. doi: 10.1186/s13287-017-0639-6 (PMC5627404; doi:10.1186/s13287-017-0639-6)
Supplement: Supplementary file 2 — Presenting flow cytometry results. (DOCX 17 kb) [file 13287_2017_639_MOESM2_ESM.docx]

**In Vitro Characterization of Canine Multipotent Stromal Cells Isolated from Synovium, Bone Marrow, and Adipose Tissue: A Donor-Matched Comparative Study**

**Names of Authors:**

Robert N. Bearden- lead author

Department of Small Animal Clinical Sciences, College of Veterinary Medicine and Biomedical Sciences, Texas A&M University, College Station, TX.

Email: rbearden@cvm.tamu.edu

Shannon S. Huggins

Department of Small Animal Clinical Sciences, College of Veterinary Medicine and Biomedical Sciences, Texas A&M University, College Station, TX.

Email: shuggins@cvm.tamu.edu

Kevin J. Cummings

Department of Veterinary Integrative Biosciences, College of Veterinary Medicine and Biomedical Sciences, Texas A&M University, College Station, TX.

Email: kcummings@cvm.tamu.edu

Roger Smith

Department of Veterinary Pathobiology, College of Veterinary Medicine and Biomedical Sciences, Texas A&M University, College Station, TX.

Email: rosmith@cvm.tamu.edu

Carl A. Gregory

Department of Molecular & Cellular Medicine, Institute for Regenerative Medicine, College of Medicine, Texas A&M University, Temple, TX.

Email: cgregory@medicine.tamhsc.edu

William B. Saunders- corresponding author

Department of Small Animal Clinical Sciences, College of Veterinary Medicine and Biomedical Sciences, Texas A&M University, College Station, TX.

Email: bsaunders@cvm.tamu.edu

**Additional File 2:**

**Table S1:** *Flow cytometry results (percentage of positive cells) for synovium, marrow, and adipose cMSCs.*

|  | | | | | | | |
| --- | --- | --- | --- | --- | --- | --- | --- |
| Source | CD9 | CD44 | CD90 | CD105 | CD34 | CD45 | STRO-1 |
| Synovium | 99.88 ± 0.11 | 97.16 ± 3.32 | 91.94 ± 9.81 | 46.16 ± 21.78 | 0.34 ± 0.27 | 0.29 ± 0.22 | 0.01 ±  1.55 |
| Marrow | 99.86 ± 0.26 | 96.21 ± 4.25 | 97.17 ± 4.55 | 17.12 ± 8.86 | 0.30 ± 0.13 | 0.23 ± 0.12 | 0.26 ±  1.14 |
| Adipose | 99.98 ± 0.05 | 96.49 ± 4.88 | 93.99 ± 8.99 | 59.84 ± 16.57 | 0.33 ± 0.26 | 0.27 ± 0.26 | 0.00 ±  1.1 |

**Table S1:** *Flow cytometry results (percentage of positive cells) for synovium, marrow, and adipose cMSCs.* Percentage positive cells reported as mean ± SD for synovium, marrow, and adipose cMSCs isolated from five canine donors.

**Figure S1 Legend:** *Immunomodulation of murine IL-6 by synovium, marrow, and adipose cMSCs.* Passage 2 cMSCs (1x10^3^–50x10^3^) were co-cultured with 1x10^4^ murine macrophage cells in CCM (n=3 wells/condition). After 24hrs, LPS (0.5µg/mL) was added to co-cultures to activate macrophages and to assess cMSCs immunomodulation. After 18hrs in LPS stimulated co-culture, media were collected and ELISA performed to determine the concentration of secreted murine IL-6. **A)** Representative murine IL-6 concentrations (mean ± SD) for an individual donor. RAW + LPS denotes IL-6 concentration from murine macrophages (RAW cells) in the absence of cMSCs (positive control). **B)** Data from panel A were transformed to reflect the percentage change in IL-6 relative to the RAW + LPS positive control in preparation for comparative analysis across all 15 cMSC preparations and are reported as mean ± SD. **C)** Scatter plots demonstrating the percentage change in IL-6 concentration relative to positive control for all 15 cMSC preparations, organized by tissue number of cMSCs present within co-cultures. Each data point represents the relative murine IL-6 detected for an individual cell preparation and “dose” of cMSCs (bar=mean across 5 donors). For all three tissues, IL-6 concentrations significantly increased in response to increasing number of co-cultured cMSCs. While cMSCs resulted in a dose-dependent increase in measured murine IL-6, adipose cMSCs had the largest effect. Asterisks denote significant differences between numbers of co-cultured cMSCs: (**) p<0.01, (***) p<0.001. Letters a and b denote significant differences in IL-6 concentrations (p<0.05).
