# Supplementary figures and images for: In-vitro characterization of canine multipotent stromal cells isolated from synovium, bone marrow, and adipose tissue: a donor-matched comparative study
Source: Stem Cell Res Ther. 2017 Oct 3;8:218. doi: 10.1186/s13287-017-0639-6 (PMC5627404; doi:10.1186/s13287-017-0639-6)

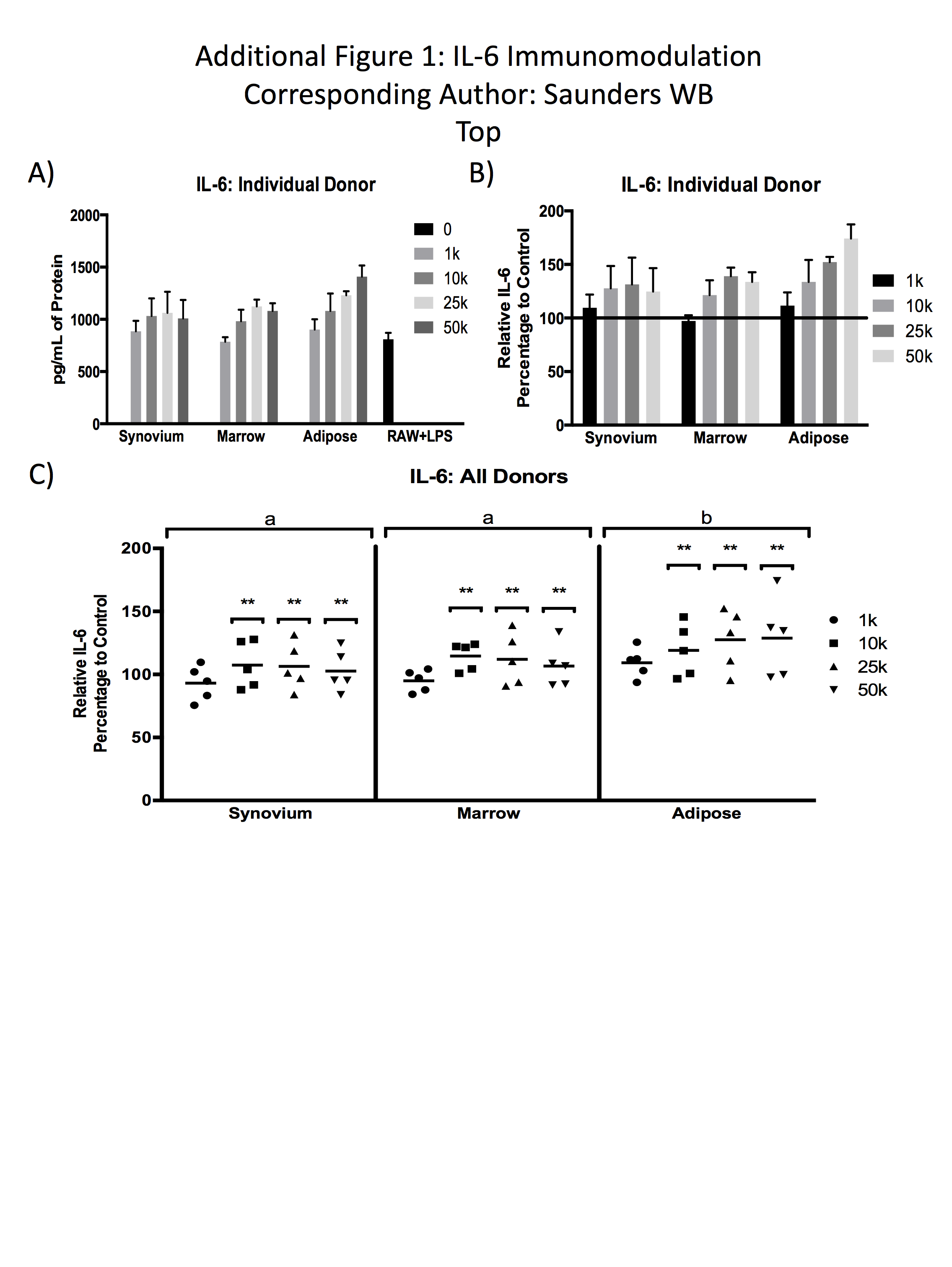

Supplement: Supplementary file 3 — Showing immunomodulation of murine IL-6. (TIFF 427 kb) [file 13287_2017_639_MOESM3_ESM.tiff]
